# Alisol A ameliorates vascular cognitive impairment via AMPK/NAMPT/SIRT1-mediated regulation of cholesterol and autophagy

**DOI:** 10.7150/thno.112661

**Published:** 2025-08-22

**Authors:** Ping Xu, Wen Zhou, Shida Wang, Linjiao Wang, Yu Bai, Shan Xing, Wenda Xue, Meng Li, Jun Shi, Haoxin Wu

**Affiliations:** 1 Nanjing University of Chinese Medicine, Nanjing 210023, Jiangsu, P. R. China.; 2 Key Laboratory of Integrative Biomedicine for Brain Diseases, School of Chinese Medicine, Nanjing University of Chinese Medicine, Nanjing, 210023, P. R. China.; 3 Union laboratory of Traditional Chinese medicine for brain science and gerontology, Nanjing University of Chinese Medicine, Nanjing, 210023, P. R. China.

**Keywords:** Alisol A, atherosclerosis-related vascular cognitive impairment, brain cholesterol homeostasis, autophagy

## Abstract

Atherosclerosis-related vascular cognitive impairment (VCI) is associated with dysregulated cholesterol metabolism and impaired autophagy. Alisol A, a natural tetracyclic triterpenoid derived from the traditional ZeXieYin Formula, has demonstrated anti-atherosclerotic and neuroprotective effects. However, its role in modulating brain cholesterol homeostasis and mitophagy in VCI remains largely unexplored.

**Methods:** To elucidate the mechanism of Alisol A and evaluate its translational relevance, we employed an *Ldlr^-/-^* mouse model of VCI induced by a high-fat diet and left common carotid artery ligation. Alisol A was administered intragastrically, and cognitive function was assessed using the Morris water maze, Y-maze, and novel object recognition tests. To probe the role of NAMPT, pharmacological inhibition and lentiviral overexpression strategies were applied. Mechanistic investigations included Western blotting, immunofluorescence, and transmission electron microscopy to examine cholesterol metabolism, oxidative stress, mitophagy, and synaptic plasticity. Additionally, molecular docking, surface plasmon resonance, and lipidomic profiling were used to explore Alisol A-NAMPT binding and downstream regulatory pathways.

**Results:** Alisol A significantly ameliorated cognitive impairment in *Ldlr^-/-^* mice. Mechanistically, it restored cholesterol homeostasis by activating the AMPK/NAMPT/SIRT1 signaling axis, upregulated UCP2 to suppress oxidative stress, and inhibited glial activation, thereby preserving neuronal structure and function. Additionally, Alisol A reactivated mitophagic flux by enhancing PINK1/PARKIN signaling and facilitating the clearance of damaged mitochondria, ultimately improving mitochondrial function. NAMPT was identified as a key molecular target mediating these neuroprotective effects.

**Conclusion:** Alisol A confers neuroprotection in VCI by regulating cholesterol metabolism, attenuating oxidative stress, and restoring mitophagy via NAMPT-mediated signaling. These findings highlight its therapeutic potential in atherosclerosis-related cognitive decline.

## Introduction

Atherosclerosis (AS) is the most common form of cardiovascular and cerebrovascular disease. Its principal pathogenic mechanisms are lipid accumulation and large-artery inflammation, which ultimately give rise to clinical complications such as myocardial infarction, stroke, and cognitive impairment. Vascular cognitive impairment (VCI) encompasses the full spectrum of cognitive decline resulting from cerebrovascular pathology including cerebral infarction, hemorrhage, small-vessel disease, or chronic cerebral hypoperfusion ranging from mild subjective cognitive complaints or mild VCI to severe deficits that meet diagnostic criteria for vascular dementia (VaD) 1. In VCI, patients exhibit only subtle declines in executive function or memory, with daily living abilities largely preserved; impairments are detected only on neuropsychological testing. By contrast, VaD arises from multiple or extensive vascular lesions, causing significant deficits across several cognitive domains and markedly impairing independent living and social function. These deficits meet the DSM-5 criteria for “major neurocognitive disorder” or the ICD-11 criteria for “dementia” 2, 3. Atherosclerosis is significantly and independently associated with cognitive decline, including impairments in memory, executive function, and language. Notably, this association remains robust even after adjusting for traditional vascular risk factors such as hypertension and diabetes, suggesting that its underlying pathological mechanisms may extend beyond conventional vascular risk scores 4. Dyslipidemia first promotes atherosclerosis, leading to stenosis and plaque formation in large and medium-sized arteries. Plaque rupture or arterial narrowing then induces downstream small-artery (penetrating artery) pathology, which progresses to cerebral small-vessel disease. Ultimately, the combined effects of large-vessel damage and small-vessel pathology result in vascular cognitive impairment. Specifically, dyslipidemia promotes lipid accumulation in large and medium-sized arterial walls by increasing circulating low-density lipoprotein (LDL) and oxidized low-density lipoprotein (ox-LDL) 5. ox-LDL activates macrophage scavenger receptors, induces monocyte infiltration, and promotes foam cell formation, ultimately contributing to the development of atherosclerotic plaques in the carotid and cerebral arteries 6, 7. These plaques may cause luminal narrowing or detach as unstable plaques, leading to cortical infarction 8. In addition, plaques can extend to the origins of perforating arteries, where they cause localized occlusion or hypoperfusion. This initiates downstream arteriolar hyalinosis and chronic hypoperfusion, manifesting as lacunar infarctions and white matter hyperintensities, which are indicative of cerebral small vessel disease (CSVD) 7. AS and CSVD act synergistically at the pathological level: large-artery stenosis exacerbates cortical ischemia, while small-vessel lesions chronically impair white matter integrity, disrupt neurovascular coupling, and compromise blood brain barrier (BBB) function 9. These pathologies characterized by white matter rarefaction, microbleeds, and accumulation of lacunes collectively form the neuropathological basis of VCI. From a pathophysiological perspective, this process represents a cascade of multisystemic injury. It is initiated by lipid deposition and inflammatory activation that lead to vascular endothelial dysfunction. Subsequently, ox-LDL stimulates monocyte infiltration and foam cell formation through activation of scavenger receptors, resulting in plaque development and arterial lumen narrowing 6, 7, 9. This narrowing impairs both cortical and deep cerebral perfusion, causing chronic hypoperfusion. Arteriolar hyalinosis and basement membrane thickening further compromise microcirculatory function. The combined effects of neuronal hypoperfusion and increased BBB permeability ultimately disrupt the neurovascular unit. Neuroimaging studies have also confirmed that patients with atherosclerosis frequently exhibit white matter rarefaction, lacunar infarctions, and cerebral microbleeds. Among these, reduced integrity of white matter tracts is strongly correlated with deficits in executive function. Epidemiological studies indicate that VaD accounts for at least 20% of all dementia cases 10, 11. Since VaD is the only form of dementia in which cognitive decline can be halted or reversed, investigating the pathophysiological mechanisms and therapeutic strategies for VCI is of great clinical importance and societal value.

In the nervous system, cholesterol is not only a fundamental structural component of neuronal membranes, contributing to membrane fluidity and stability, but also plays a critical role in neurotransmitter synthesis, synapse formation, maintenance, and signal transmission 12, 13. Numerous epidemiological studies have established that elevated low-density lipoprotein cholesterol (LDL-c) levels are associated with an increased risk of cognitive impairment 14, 15. A large cohort study by Iwagami et al, involving over 1.8 million participants, reported that midlife LDL-c levels were associated with a higher incidence of dementia more than a decade later 16. Additional evidence from the Atherosclerosis Risk in Communities (ARIC) study suggested that individuals with LDL-c below 100 mg/dL experienced slower cognitive decline over 20 years 17. In hypercholesterolemia, excessive cholesterol triggers NOD-like receptor family pyrin domain containing 3 (NLRP3) inflammasome activation and microglial-mediated neuroinflammation 18, 19, leading to amyloid-β (Aβ) accumulation, oxidative stress 20, 21, and neuronal pyroptosis 22, 23. Additionally, cholesterol imbalance alters the crosstalk between astrocytes and neurons, further amplifying the inflammatory response 24. Elevated cholesterol levels have been shown to enhance acetylcholinesterase activity, resulting in reduced acetylcholine availability and impaired synaptic transmission and cognitive processing 25, 26. Cholesterol dysregulation serves as a critical molecular link between atherosclerosis and cognitive impairment 27. Increased circulating total cholesterol and low-density lipoprotein cholesterol accelerate atherosclerotic plaque formation, while their metabolic byproducts, such as oxysterols, can cross the BBB and disturb cerebral cholesterol homeostasis via activation of liver X receptor (LXR) signaling 19, 28, 29. In ApoEε4 carriers, astrocytes exhibit a tendency toward abnormal lipid accumulation, which compromises Aβ clearance efficiency 30, 31. Animal studies have demonstrated that a high-cholesterol diet enhances hippocampal Aβ₄₂ deposition and downregulates synaptophysin expression in APP/PS1 transgenic mice, suggesting that disrupted lipid metabolism may directly impair synaptic integrity through the promotion of Aβ aggregation 32-34.

Mitophagy is crucial for maintaining mitochondrial homeostasis by selectively degrading damaged mitochondria 35 to regulate mitochondrial number in response to metabolic demands 36. Notably, mitophagy is pivotal for neuronal function and survival by sustaining a healthy mitochondrial, thus preventing neuronal death 37, 38. Enhancing mitophagy can help with neurological issues 39, and an autophagy inducer has been shown to protect the brains of mice 40, 41. Improving mitochondrial dysfunction can improve cognitive deficits through the PINK1 pathway 42. In parallel, autophagy has gained attention for its role in lipid turnover and mitochondrial quality control 38. While not unique to AS-related VCI, dysregulated autophagy-particularly mitophagy-may exacerbate neuronal dysfunction in cholesterol-induced brain injury 43, 44. Moreover, studies have shown that autophagy facilitates cholesterol clearance in microglia and macrophages 45, whereas its inhibition leads to lipid accumulation and foam cell formation 46, 47. These findings underscore autophagy's role as a potential therapeutic target in AS-related VCI 47, 48. In this context, previous research has demonstrated that Alisol A can induce autophagy in a dose-dependent manner 49-51. Thus, modulation of autophagy has emerged as a potential therapeutic mechanism underlying the protective effects of Alisol A against AS-related VCI.

The AMP-activated protein kinase (AMPK)/nicotinamide phosphoribosyltransferase (NAMPT)/sirtuin 1 (SIRT1) axis plays a critical role in regulating key pathways associated with cellular aging, energy metabolism, and neuroprotection 52. Disruption of this axis impairs autophagy and exacerbates metabolic stress 53, 54. Notably, under hyperlipidemic conditions, disturbances in lipid metabolism can inhibit the activity of AMPK/NAMPT/SIRT1 signaling 55, leading to reduced autophagic flux. NAMPT plays a critical role in cellular energy homeostasis and mitophagy by regulating NAD⁺ biosynthesis, which is essential for the activation of sirt1, a key deacetylase involved in mitochondrial function and metabolic regulation 56. As an upstream energy sensor, AMPK initiates energy-stress responses, while NAMPT serves as a rate-limiting enzyme in NAD⁺ regeneration and provides essential metabolic support for AMPK-dependent activation of sirt1. Studies in animal models have revealed that *Nampt* deficiency in neurons leads to hippocampal dysfunction and memory loss 57, 58. Supplementation with nicotinamide mononucleotide (NMN), a NAMPT-related NAD⁺ precursor, enhances cerebrovascular function and reduces Aβ burden in the brain 59. These findings highlight NAMPT as a promising molecular target in AS-related VCI 60. In the context of AS-related VCI, dysregulation of the AMPK/NAMPT/SIRT1 signaling pathway may impair mitophagy, thereby exacerbating cognitive decline. Therefore, investigating the role of Alisol A in modulating the AMPK/NAMPT/SIRT1 pathway and mitophagy may offer novel mechanistic insights and therapeutic potential for the treatment of AS-related VCI.

ZeXieYin Formula, a classical prescription in traditional Chinese medicine (TCM), has been widely applied in clinical settings for centuries. The herbs included in this formula are rich in bioactive compounds with known metabolic regulatory, anti-inflammatory, and antioxidant properties. In our previous studies, ZeXieYin Formula demonstrated significant therapeutic effects on cognitive impairment induced by high-fat diet related atherosclerosis, with the following key findings: (1) ZeXieYin Formula alleviated neuroinflammation and reduced Aβ deposition by modulating the MAPK/NF-κB signaling pathway in the hippocampus, thereby enhancing synaptic plasticity and improving cognitive function 61; (2) It promoted hippocampal neurogenesis and synaptic plasticity through regulation of the CaMKII-AMPA receptor pathway, thereby improving cognitive performance in an Alzheimer's disease mouse model 62; (3) The decoction also counteracted the synaptic damage pathway mediated by the gut microbial metabolite trimethylamine N-oxide (TMAO), alleviating its negative impact on neuronal plasticity and enhancing learning and memory capacity 63. From a formula-based perspective, the individual herbs in ZeXieYin Formula possess notable neuroprotective potential. For example, Pyrola calliantha (Lu Xian Cao) has been shown to suppress the release of proinflammatory cytokines, mitigate glial cell mediated neurotoxicity, promote neurogenesis, and enhance the expression of synaptic proteins beneficial for maintaining neuronal plasticity and function 64. In our prior work, we applied UPLC-MS/MS and other analytical techniques to establish a chemical fingerprint of the aqueous extract of ZeXieYin Formula and conducted quantitative analysis of representative compounds across multiple batches 65, 66. The results revealed that Alisol A, a triterpenoid compound, was consistently the most abundant bioactive constituent, with its content stable across batches and meeting the extraction quality standards specified by the Chinese Pharmacopoeia. Derived from Rhizoma Alismatis, a traditional Chinese herb with known lipid-lowering and cardioprotective effects, Alisol A has attracted attention for its potential therapeutic roles 67-77. Recent pharmacological studies have shown that Alisol A can reduce lipid levels, cross the blood-brain barrier, and improve cognition in high-fat diet-induced models of brain aging 78.

Notably, Alisol A may exert its effects through sirt1 activation, although the mechanisms remain to be fully elucidated 69, 77-80. Whether Alisol A modulates cholesterol metabolism and mitophagy via the AMPK/NAMPT/SIRT1 pathway in VCI has not been investigated 81. Although the precise mechanisms of Alisol A remain insufficiently characterized, it has demonstrated promising effects in metabolic regulation and neuroprotection, suggesting high research value. Therefore, in this study, we selected Alisol A as a representative active component to further investigate its mechanistic role in a model of VCI. This study aims to elucidate the mechanisms by which Alisol A targets AMPK/NAMPT/SIRT1 to coordinately regulate cerebral cholesterol metabolism and PINK1/PARKIN-mediated mitophagy, thereby restoring synaptic plasticity and improving cognitive function. By uncovering these pathways, the findings provide both a novel molecular target and a mechanistic foundation for the development of therapeutic strategies against AS related VCI.

## Materials and Methods

### Model building and drug intervention

Five-week-old male C57BL/6 J mice and B6/JGpt-*Ldlr*^em1Cd82^/Gpt (*Ldlr^-/-^*) male mice of the same age were procured from Gem Pharmatech Co., Ltd in Nanjing, China. Mice were housed in groups of three to five animals per cage under controlled environmental conditions (temperature: 22 ± 2 °C; 12 h light/dark cycle). All mice are randomly divided into groups, with 15 mice in each group. Body weights were recorded weekly throughout the study. This study used male mice to avoid potential effects associated with the estrous cycle 82, 83. At 8 weeks of age, mice were subjected to left common carotid artery ligation under general anesthesia. Following induction with isoflurane (3% for induction, 1.5% for maintenance), the left common carotid artery was carefully isolated through a midline cervical incision and permanently ligated using 6-0 silk sutures 84, 85. After the surgery is completed, the incision is sutured, and the mouse is then returned to the cage to recover. Buprenorphine (0.1 mg/kg, subcutaneous) was administered for postoperative analgesia. Mice were healthy and housed under SPF-grade conditions. The relative humidity was 50% to 60% throughout the experiment. All animals had at least 7 days to adjust to their living conditions. *Ldlr^-/-^* mice were continuously fed a high-fat diet containing 1.25% cholesterol and 20% fat. A high-fat diet was purchased from Jiangsu Synergetic Pharmaceutical Bioengineering Co, Ltd. The control group received standard chow and normal saline intragastrically. Behavioral tests were performed at 18 weeks of age. If mice failed to reach the criteria within the behavioral tests, they were eliminated from the study. Continue the experiment with the successfully modeled mice. The criteria for inclusion were as follows: During the spatial exploration experiment, the mouse spent less than 20 s in the target quadrant. The Y-maze task had a spontaneous alternation rate below 30%. The novel object recognition test showed a novel object recognition index under 40%.

Alisol A (19885-10-0; MedChemExpress, Shanghai, China) was administered intragastrically (i.g.) at 15 mg/kg or 30 mg/kg once daily for 2 months 81. All animals were administered the treatment via gavage, with the control group receiving an equal volume of saline. NAMPT inhibitor: FK866 (658084-64-1, MedChemExpress, Shanghai, China) administered at a dose of 25 mg/kg once daily for 2 months via intraperitoneal injection 86, 87. NMN (1094-61-7, MedChemExpress, Shanghai, China, 100 mg/kg, i.g.) once daily for 2 months 88. The experiment received approval from the Ethical Committee on Animal Experimentation at Nanjing University of Chinese Medicine.

### Cognitive and behavioral tests

The Morris water maze (MWM) utilized an artificially partitioned pool with four quadrants and a submerged platform hidden beneath the water's surface. Following an initial day of adaptability training, mice underwent formal training for five consecutive days, culminating in a positioning navigation test on the sixth day. Subsequently, the platform was taken out, allowing mice to swim freely in the pool for 60 s. The time spent in the target quadrant was recorded for analysis.

In the Y-maze experiment, mice were subjected to a custom-made apparatus consisting of three symmetrical arms (35 cm × 10 cm × 20 cm), each positioned at a 120° angle. During a 5 min trial, mice explored two arms of the maze while the third arm was blocked. The maze was cleaned before each trial to remove any lingering odors. Subsequent to the training trial, a single 5 min test trial was conducted, during which spontaneous alternations and arm entries were meticulously recorded. The alternation percentage was determined by dividing the number of alternations by the total number of arm entries and multiplying the result by 100%.

In the Novel Object Recognition (NOR) task, each mouse underwent a familiarization phase in which they were placed in a box and exposed to two identical objects for a duration of 5 min. To prevent the influence of olfactory cues, the objects were meticulously cleaned between trials. The subsequent day, the mouse was reintroduced to the same box, but one of the familiar objects was replaced with a novel object of distinct color and shape. The exploration time of each object was quantified using the ANY-maze video-tracking system, with a defined 2 cm^2^ area surrounding the objects to record nose entries as indicators of object exploration.

### Model evaluation

Hematoxylin-eosin Staining (HE): Mice were sedated with sodium pentobarbital, euthanized by cervical dislocation, and their tissues were promptly dissected on ice. Five mice from each group were randomly chosen for fixation in 4% paraformaldehyde at 4 °C for 24 h. The remaining tissues were dissected on ice, snap-frozen, and stored at -80 °C. The samples were embedded in paraffin and made into tissue sections. After HE staining, sections were observed under an optical microscope and photographed.

Oil Red O Staining (ORO): The tissue was frozen quickly in cutting temperature compound (OCT) compound and cut into 10 µm sections. Frozen sections were applied for ORO. Counterstaining was performed using hematoxylin. Following sealing, the slices were examined using a light microscope and imaged. Plaque and vessel measurements were taken using Image J software. Plaque stenosis was determined by comparing plaque area to total vessel area, and lipid content was calculated as the percentage of ORO area in relation to total plaque area.

Serum biochemical analysis: Serum triglycerides (TG), total cholesterol (TC), high-density lipoprotein cholesterol (HDL-c), and LDL-c levels were measured using enzymatic kits from Lei Du, Shenzhen, China, and an Automatic Biochemical Analyzer (Chemray240) as per kit instructions.

For determination of cholesterol in mouse brain tissue, a Total Cholesterol Assay Kit (A111-1-1; Nanjing Jiancheng Bioengineering Institute, Nanjing, China) was used. Brain samples were accurately weighed and homogenized in nine volumes of homogenization buffer (w/v, g: mL = 1:9) on ice using a mechanical homogenizer. Following the manufacturer's instructions, the homogenate was thoroughly mixed and then incubated at 37 °C for 10 min. Absorbance was measured at 500 nm with a microplate reader.

### Hippocampal neurons morphology and function

Transmission electron microscopy (TEM): Each hippocampal tissue was chopped into small pieces using sterile surgical blades (about 0.5-1 mm^3^ in size). Five specimens from each group were fixed in 2.5% glutaraldehyde solution and prepared for evaluation using TEM. Ultrathin sections were made and images were captured with a Hitachi H-7500 transmission electron microscope at a magnification of 4000-8000 ×. The images were analyzed and measured using Image-pro plus 6.0.

Pathological examination: The brains of mice were fixed in paraformaldehyde after anesthesia and perfusion, then cut into 5 µm slices for examination. Sections were then processed for Nissl Staining (NS) or immunohistochemistry (IHC). Optical microscopy was used to observe and capture images.

Immunofluorescence (IF): Sections were blocked with 3% BSA in PBS for 30 min at room temperature, then incubated with primary antibodies overnight at 4 °C. After rinsing with cold PBS, secondary antibodies were applied for 1 h at room temperature. Counterstaining was done with DAPI, and images were captured and analyzed using Digital Pathology system and Viewer software.

Golgi staining: Experimental animals were injected with anesthesia, then the skin on the top of the mouse skull was cut open to carefully remove the brain. The brain was washed with saline to remove blood stains and then immersed in fixative. Use a shaking slicer for tissue sectioning, with a thickness of 100-200 μm. Capture images with a panoramic digital slice scanning microscope (VS120) and subsequently conduct analysis utilizing Fiji software.

The studies used the following antibodies. Anti-GFAP antibody (GB12096, Servicebio, 1:500), anti-IBA-1 antibody (GB12105, Servicebio, 1:500). anti-PSD95 antibody (20665-1-AP, Proteintech, 1:300), anti-SYNAPSIN-1 antibody (20258-1-AP, Proteintech, 1:300), anti-UCP2 antibody (GB11377, Servicebio, 1:300), anti-CD68 antibody (GB113109, Servicebio, 1:300), anti-α-SMA antibody (GB111364, Servicebio, 1:300), anti-HMGCR antibody (DF6518, Affinity, 1:200), anti-ABCA1 Polyclonal antibody (26564-1-AP, Proteintech, 1:300). anti-NAMPT/PBEF Polyclonal antibody (11776-1-AP, Proteintech, 1:300), anti-SIRT1 antibody (13161-1-AP, Proteintech, 1:300). anti-CYP46A1 Polyclonal antibody (124861-1-AP, Proteintech, 1:300). anti-OPTN antibody (GB114324, Servicebio, 1:300), anti-TOM20 antibody (GB111481, Servicebio, 1:300), anti-NMDAR2A/GRIN2A Polyclonal antibody (28525-1-AP, Proteintech, 1:300), anti-NMDA2B antibody (21920-1-AP, Proteintech, 1:300).

### Mendelian randomization analysis

Using Mendelian randomization to explore the causal link between coronary atherosclerosis and vascular dementia. The dataset used comes from the IEU Open genome-wide association studies (GWAS) Project. The analysis was performed using the Two Sample MR package (v0.5.7) in R software (v4.3.1). Note: 'Exposure' refers to the exposure data number; 'Outcome' denotes the outcome data number; 'Method' specifies the analysis method employed; 'Q' represents the heterogeneity test Q statistic; 'Q_def' indicates the degrees of freedom associated with the Q statistic; 'Q_pval' is the p-value of the Q statistic, which assesses the statistical significance of the heterogeneity test results. A p-value less than the predetermined significance level (commonly 0.05) suggests the presence of heterogeneity within the data 89. In the analysis of GWAS, coronary atherosclerosis (finn_b_I9_CORATHER) was considered as the exposure factor, vascular dementia (finn_b_F5_VASCDEM) is served as the outcome factors. Mendelian randomization analysis was performed utilizing various regression models, including the inverse variance weighted (IVW) method, MR Egger method, weighted median method.

### Serum lipidomics

The collection and use of human serum samples were approved by Ethics Review Committee of Nanjing Hospital of Chinese Medicine (Ethics Review No. KY2023091). All procedures were performed in strict compliance with the Regulations of Ethical Review of Biomedical Research Involving Human Subjects, the Declaration of Helsinki, and the International Ethical Guidelines for Biomedical Research Involving Human Subjects. Inclusion criteria for clinical serum samples from patients diagnosed with hyperlipidemia are as follows: (1) Demographic parameters: Participants must be aged between 20 and 50 years, with an equal distribution of genders to ensure sample balance. (2) Medical history: Participants should have a confirmed diagnosis of atherosclerosis, while individuals with other metabolic disorders (e.g., diabetes, thyroid disease) are excluded to minimize confounding variables. (3) Biochemical Indicators: The diagnosis of hyperlipidemia is dependent on serum biochemical markers, including cholesterol and triglyceride concentrations. (4) Complications: To maintain the focus of the study and ensure the reliability of the results, individuals with severe complications, such as liver or kidney disease, are excluded. (5) Medication Use: The use of medications by patients is a critical consideration. This study will exclude individuals currently taking drugs that influence lipid metabolism, such as specific steroids or antidepressants, to prevent confounding effects on the outcomes. (6) To avoid individual differences, 4-5 samples were pooled together into one sample for analysis.

Samples were thawed on ice, and metabolites were extracted with a lipid extraction buffer. Each 20 μL sample was mixed with 120 μL of precooled buffer (IPA: ACN: H_2_O = 2:1:1), vortexed for 1 min, incubated at room temperature for 10 min, and stored overnight at -20 °C. After centrifuging at 4, 000 g for 20 min, supernatants were transferred to new 96-well plates and stored at -80 °C for LC-MS analysis. Pooled QC samples were prepared by combining 10 μL from each extraction mixture.

Samples were processed with an LC-MS system following machine instructions. Chromatographic separations used an ACQUITY UPLC System and a Kinetex UPLC C18 column, maintained at 55 °C with a 0.3 ml/min flow rate. The mobile phase comprised solvent A (60% ACN, 40% H_2_O, 0.1% formic acid) and solvent B (90% IPA, 10% ACN, 0.1% formic acid). Gradient elution: 0-0.4 min at 30% B; 0.4-1 min from 30% to 45% B; 1-3 min from 45% to 60% B; 3.5-5 min from 60% to 75% B; 5-7 min from 75% to 90% B; 7-8.5 min from 90% to 100% B; 8.5-8.6 min at 100% B; 8.6-8.61 min from 100% to 30% B; 8.61-10 min at 30% B.

A high-resolution TripleTOF6600 mass spectrometer (SCIEX) operated in both positive and negative ion modes with curtain gas at 30 PSI, ion source gases at 60 PSI, and an interface heater at 650 °C. The Ionspray voltage was 5000 V for positive mode and -4500 V for negative mode. Data were collected in IDA mode with a TOF mass range of 60-1200 Da. Survey scans lasted 150 ms, capturing up to 12 product ion scans if they exceeded 100 counts/s and had a 1 + charge state. The cycle time was 0.56 s, using four 11 kHz time bins, monitored by a 40 GHz multichannel TDC detector with four-anode/channel detection. Dynamic exclusion lasted 4 s, and mass accuracy was calibrated every 20 samples. A pooled quality control sample was run after every 10 samples to ensure LC-MS stability.

MS data pretreatments, such as peak picking, grouping, retention time correction, and isotope/adduct annotation, were conducted with XCMS software. LC-MS raw data were converted to mzXML and processed using XCMS, CAMERA, and metaX in R, identifying ions by retention time and m/z to create a 3D matrix of peak indices, sample names, and ion intensities. Metabolites were matched to KEGG and HMDB databases within a 10 ppm mass difference, with molecular formulas confirmed by isotopic distribution and validated using an in-house fragment spectrum library.

Peak data intensity was processed with metaX, removing features found in less than 50% of QC samples or 80% of biological samples. Missing data were imputed using the k-nearest neighbor method. PCA was used to detect outliers and assess batch effects. A robust LOESS correction was applied to QC data to minimize signal drift over time, and features with over 30% relative standard deviation in QC samples were excluded.

Student t-tests identified differences in metabolite concentrations between two phenotypes, with P values adjusted for multiple comparisons using FDR. Supervised PLS-DA via metaX distinguished variables between groups, considering features with a VIP value above 1.0 as important.

### Gene Expression Omnibus analysis

Gene Expression Omnibus (GEO) Dataset: The dataset designated as GSE100927 was retrieved from the GEO database, accessible at http://www.ncbi.nlm.nih.gov/geo. Atherosclerotic lesions, along with control arteries devoid of such lesions, were procured from the carotid, femoral, and infra-popliteal arteries of deceased organ donors. Subsequently, RNA was extracted from these samples and subjected to hybridization on microarrays. All datasets were obtained from the GEO database, with the data downloaded in MINiML format, encompassing all platforms, samples, and comprehensive GSE records within the GSE. For datasets that were not pre-normalized, a log2 transformation was uniformly applied. To address batch effects across various subsets within the same dataset and platform, we employed the `removeBatchEffect` function from the `limma` package in R. This transcriptomic analysis focused on human peripheral arteries, encompassing the carotid, femoral, and infra-popliteal regions, in both atherosclerotic and control tissues.

### Molecular dynamics simulations

We used Gromacs2020 Molecular dynamics simulations (MD) simulations to investigate NAMPT binding to Alisol-A, using the AMBER99SB-ILDN forcefield for proteins and the GAFF forcefield for ligands. Sobtop was used to establish the GAFF force field. The restrained electrostatic potential (RESP) method26 was then used for charge fitting. The TIP3P water model was used for the explicit waters. The distance between the edge of the box and the solute atom was established at a minimum of 1.0 nm, with the addition of sodium or chloride ions as necessary to achieve charge neutrality within the system. The molecular dynamics simulation process encompassed four key stages: minimization, heating, equilibration, and production run. The ligand-protein complexes were used to calculate the binding free energy.

### Surface plasmon resonance

Surface plasmon resonance (SPR): The aim of this study was to evaluate the binding affinity between NAMPT and Alisol A utilizing SPR technology. For chip preparation, an activator solution was formulated by combining 400 mM EDC and 100 mM NHS immediately before injection. The CM5 sensor chip was activated for 420 s using this mixture at a flow rate of 10 μL/min. Ligand immobilization was performed by diluting NAMPT to a concentration of 20 μg/mL in an immobilization buffer, followed by injection into the sample channel Fc2 at a flow rate of 10 μL/min, typically achieving immobilization levels of approximately 14,700 RU. It is noteworthy that the reference channel Fc1 does not require a ligand immobilization step. Subsequently, the chip was deactivated with 1 M ethanolamine hydrochloride at a flow rate of 10 μL/min for 420 s. Analyte analysis was conducted using a multi-cycle method. Alisol A was diluted with the analyte buffer to achieve eight different concentrations: 100, 50, 25, 12.5, 6.25, 3.12, 1.56, and 0 μM. The Alisol A samples were injected into channels Fc1 through Fc3 at a flow rate of 20 μL/min, allowing for an association phase lasting 100 s, followed by a dissociation phase of 180 s. Both the association and dissociation phases were performed in the analyte buffer. This process was repeated for eight cycles, with analyte concentrations increasing sequentially. After each cycle of interaction analysis, the chip was regenerated.

### Cell culture

The HT22 cell line comes from immortalized mouse hippocampal neurons and was obtained from the Chinese Academy of Sciences cell bank. The culture medium was purchased from Gibco, USA. After growing to 80% confluence, the HT22 cells were used for the experiment. HT22 cells were enriched with cholesterol using β-Amyloid (25-35) (Aβ_25-35_) (131602-53-4, MedChemExpress, Shanghai, China) 81, 90 and CHO: MCD (C4951, Sigma-Aldrich) complex for 24 h. Alisol A (19885-10-0, MedChemExpress, Shanghai, China). In order to effectively deplete the intracellular NAMPT content, certain experiments necessitated a 24 h pretreatment with the NAMPT inhibitor FK866 (658084-64-1, MedChemExpress, Shanghai, China).

Primary neuron isolation. Cortical (or hippocampal) neurons were isolated from postnatal day 0-1 (P0-P1) C57BL/6, as previously described. Briefly, brain tissue was dissected, digested with papain, and triturated into single-cell suspension. Neurons were cultured in Neurobasal medium supplemented with B27, GlutaMAX, and penicillin-streptomycin. Cultures were maintained for 7-10 days *in vitro* (DIV) prior to treatment.

### Mitochondria membrane potential

Mitochondrial activity was measured using JC-1 staining (C2006, Biyuntian, China). After cell treatments, the culture medium was removed and cells were washed three times with PBS. The cells were then exposed to JC-1 working solution for a duration of 20 min at 37 °C, followed by two washes with JC-1 staining buffer, and subsequently visualized using an Olympus fluorescence microscope. Carbonyl cyanide m-chlorophenyl hydrazone (CCCP) (C2006-4, Biyuntian, China) was used to activate mitophagy. Control cells were treated with or without 10 μM CCCP for 20 min.

### NAD^+^ assay

NAD^+^ assay was carried out with Enhanced NAD^+^/NADH Assay Kit with WST-8 (S0176S, Beyotime Biotechnology, Shanghai, China). The specific operation was carried out according to the instructions. A volume 200 μl of enhanced NAD^+^/NADH extract was added using a pipette, following the ratio of 200 μl per 1 million cells. Tissue samples were rinsed with pre-cooled PBS to eliminate blood residues. Subsequently, 600 μl of enhanced NAD^+^/NADH extraction solution was added at a ratio of 200 μl per 10 mg of tissue. Finally, the NADH Standard Curve was generated. Total NAD quantification was conducted at an optical density of 450 nm, utilizing a standard curve generated from established NADH concentrations.

### ROS, SOD, GSH, and MDA

Intracellular reactive oxygen species (ROS) levels were quantified through the utilization of 2', 7'-Dichlorofluorescein diacetate (DCFH-DA, D6883, Sigma-Aldrich) fluorescence. Incubate the sample with a 1000-fold diluted solution of DCFH-DA for a duration of 1 h. The mean fluorescence intensity at an excitation wavelength of 488 nm and an emission wavelength of 535 nm was measured utilizing an Accuri C6 flow cytometer (Beckman Coulter, Brea, CA, USA). Nanjing Jiancheng assay kits were used to measure superoxide dismutase (SOD) (A001-3-2), reduced glutathione (GSH) (A006-2-1), and malondialdehyde (MDA) (A003-4-1) activity in mouse hippocampal tissues as per the manufacturer's instructions.

### Western blotting

Proteins were extracted, electrophoresed, and transferred onto a PVDF membrane. Blots were blocked with milk, incubated with primary and secondary antibodies, and washed with TBST. After dripping the developing solution on the membrane, the chemiluminescence imaging system was used for detection. Blotted bands were quantified with Image J software.

The studies used the following antibodies. GAPDH (2118, Cell Signaling Technology, 1:1000), anti-SREBP2 antibody (DF7601, Affinity, 1:1000), anti-NR1H3 Polyclonal antibody (14351-1-AP, Proteintech, 1:2000), anti-CYP46A1 antibody (12486-1-AP, Proteintech, 1:1000), anti-UCP2 antibody (89326, Cell Signaling Technology, 1:1000). anti-AMPK antibody(2532, Cell Signaling Technology, 1:1000), anti-p-AMPKα^Thr172 or Thr183^ antibody(GB114323, Servicebio, 1:1000), anti-NAMPT antibody(11776-1-AP, Proteintech, 1:1000), anti-SIRT1 antibody (13161-1-AP, Proteintech, 1:1000), anti-ACETYI-LYSINE antibody (DF7729, Affinity, 1:1000), anti-PGC1-α antibody (66369-1-1G, Proteintech, 1:1000), anti-P62/SQSTM1 antibody(T55546, Ab-mart, 1:1000), anti-BECLIN1 antibody (GB112053, Servicebio, 1:500), anti-PARKIN antibody (GB11596, Servicebio, 1:500), anti-PINK1 antibody (23274-1-AP, Proteintech, 1:1000), anti-LC3B antibody(T55992, Ab-mart, 1:1000), anti-COXIV antibody (GB11250, Servicebio, 1:500), anti-TOM20 antibody (GB111481, Servicebio, 1:500). anti-HMGCR antibody (DF6518, Affinity, 1:1000), ABCG5 antibody (DF8401, Affbiotech, 1:1000), ABCG8 antibody (DF6673, Affbiotech, 1:1000).

### siRNA was used to knock down NAMPT expression

The Small molecule interfering RNA (siRNA) was purchased from Shanghai Shenggong Biological Engineering Co., Ltd. Following the manufacturer's instructions, Lipofectamine RNAiMAX transfection reagent (13778150, ThermoFisher Scientific, USA) was used for transfection. The lyophilized siRNA was dissolved in RNase-free water to a concentration of 20 μM and aliquoted for storage at -20 °C. For the experiment, the siRNA was diluted to the required working concentration (final 50 nM) using Opti-MEM (Gibco). HT22 cells were seeded at 2 × 10⁵ cells/well in a 6-well plate, with 2 mL of complete medium per well, and cultured until the cells adhered and reached approximately 50-60% confluence. The pre-diluted siRNA was slowly added to the transfection reagent working solution, gently mixed, and incubated at room temperature for 15 min to form stable complexes. The siRNA-transfection reagent mixture was then evenly distributed into each well, and the plate was gently shaken to ensure even distribution within the wells. After 4 to 6 h of transfection, the old medium was discarded, and 2 mL of fresh complete medium was added to continue culture for 48 h. After collecting the cells, protein extraction was performed, and Western blotting was conducted to detect the expression of the target protein.

### Lentivirus-induced overexpression of NAMPT in cells

The NAMPT lentivirus used in the experiment was purchased from Shanghai Jikai Gene Biotechnology Co., Ltd. Before the experiment, cells were prepared as a single-cell suspension at a concentration of 5 × 10⁴ cells/mL and evenly seeded into a 12-well plate. When the cell confluence reached approximately 60% to 70%, the virus infection process was initiated. A virus infection system was prepared based on a multiplicity of infection (MOI) of 10. The original culture medium was then discarded, and fresh complete culture medium was added, along with the prepared infection solution. After a 12 h exposure to the virus, the medium was replaced, and the cells' growth status was monitored in real-time using a microscope. Once the cells stabilized, the culture medium was changed to complete medium containing 1 μg/mL puromycin to select for successfully infected cells, while the transfection efficiency was assessed under a fluorescence microscope. Finally, samples with optimal infection efficiency were selected for subsequent protein extraction and related experimental analysis.

### Co-immunoprecipitation

Co-Immunoprecipitation (COIP): Protein immunoprecipitation kit (Protein A/G magnetic bead method) (G2237, Servicebio). Add an appropriate amount of pre cooled IP cell lysis buffer to the cell culture dish (add protease inhibitor to the IP lysis buffer), lyse the cells at 4 °C for 10 min, and repeatedly blow with a pipette during this period. Then transfer the cell suspension to a 1.5 ml centrifuge tube and continue to lyse on ice for 20 min; Centrifuge at 12000 rpm and 4 °C for 10 min, transfer the supernatant into a new 1.5 ml centrifuge tube, operate on ice, and then measure using the BCA method; Take a small amount of supernatant and denature it for input experiments, i.e. WB detection of target proteins.

Add 1.0 μg of IgG and 20 μL of protein A/G beads (thoroughly mixed before use) to the supernatant of the negative control (IgG) group protein. The experimental group directly adds 20 μL of protein A/G beads and incubates at 4 °C for 1 h by shaking; 2000 g, 4 °C, centrifuge for 5 min, take the supernatant; Add antibodies and incubate overnight at 4 °C; Add 80 μL of protein A/G beads and incubate at 4 °C for 2 h; 4 °C, 2000 g, centrifuge for 5 min, collect immunoprecipitation complexes; Wash the immunoprecipitation complex with 1ml pre cooled IP lysis buffer 4 times, each time at 4 °C, 2000 g, and centrifuge for 5 min; After the last wash, try to absorb the supernatant as much as possible, then add 80 μL of 1 × reduction type loading buffer, boil in boiling water for 10 min, 4 °C, 1000 g, centrifuge for 5 min, and take the supernatant sample for WB detection.

Cells were treated with 1 μM acetyltransferase inhibitor - trichostatin A (TSA) (A8183, APExBIO) and 5 mM acetyltransferase activator - nicotinamide (NAM) (HY-B0150, MCE) for 12 h.

### Statistical processing

SPSS 18.0 was used for statistical analysis, reporting means ± SEM, with one-way ANOVA and LSD-t test for group comparisons.

## Results

### Alisol A improves Atherosclerosis related vascular cognitive impairment in *Ldlr^-/-^* mice

**Figure 1:**
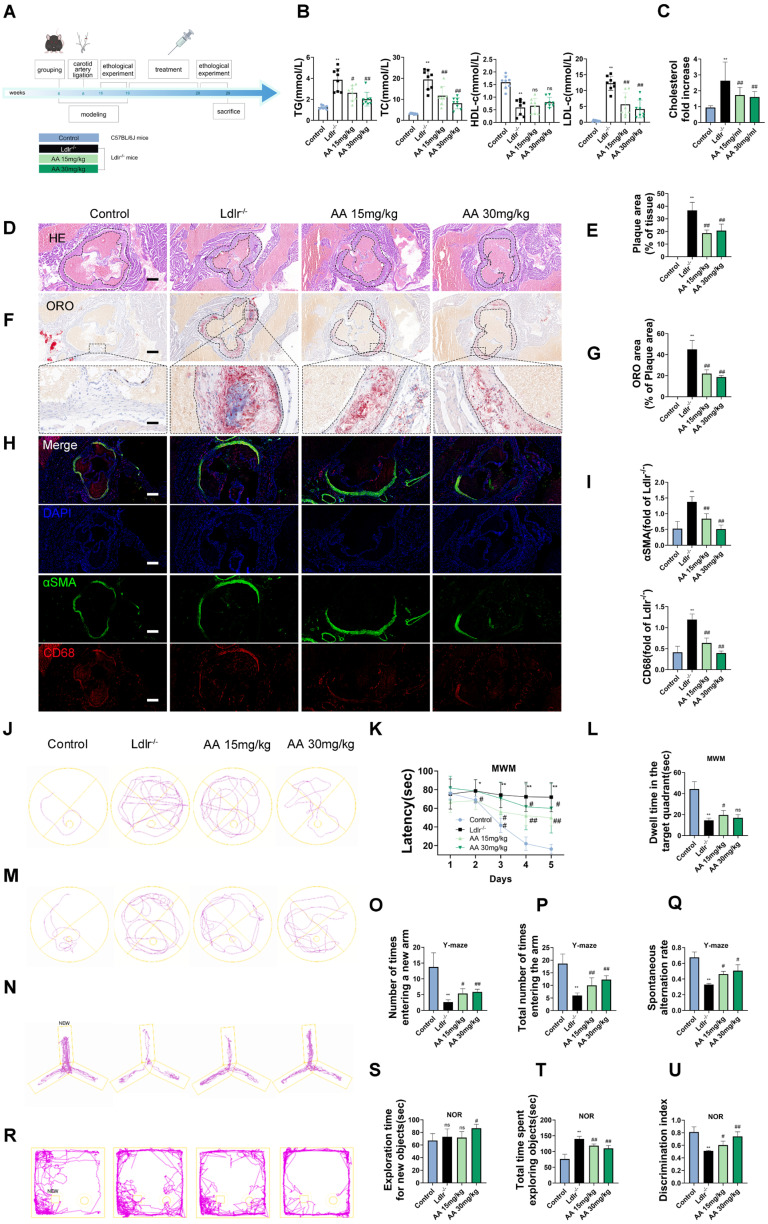
** Alisol A treatment alleviates atherosclerosis development and ameliorates cognitive function in *Ldlr^-/-^* mice. (A)** The flow diagram of the experimental design of the mice experiments; **(B)** Triglycerides (TG), serum total cholesterol (TC), high density lipoprotein cholesterol (HDL-c), low density lipoprotein cholesterol (LDL-c) levels in mice were detected by a biochemical analyzer, n = 10; Total cholesterol concentration in the brain of mouse **(C)**; HE stained **(D-E)** along with Oil Red O stained **(F-G)** of aortic root lesions, revealed the extent of atherosclerotic plaque in the aortic root, percentage of plaque area in aorta and the percentage lipid content of the aortic root plaques, scale: 100 μm, enlarge scale: 50 μm, n = 5; **(H)** IF images stained for αSMA and CD68, and quantification of expression **(I)**, scale: 100 μm, n = 5; **(J)** The MWM, the escape latency of water maze **(K)** and the mean time to stay in the target quadrant at the last experiment **(L-M)**, n = 8; **(N-O)** Y-maze, the number of total arm entries, number of entries into the open arm **(P)**, the spontaneous alternation rate in the Y-maze test **(Q)**, n = 8; **(R)** The NOR, the total object exploration time **(S)**, the time spent exploring the novel object **(T)**, The Recognition Index is operationally defined as the proportion of time allocated to exploring the novel object relative to the cumulative time spent exploring both objects **(U)**, n = 8. Compared to the control group, ^*^ P < 0.05, ^**^ P < 0.01; compared to the *Ldlr^-/-^*, ^#^* P* < 0.05, ^##^* P* < 0.01.

As shown in the mouse experimental design flowchart, *Ldlr^-/-^* mice were treated with Alisol A via gavage (Figure 1A). Compared to *Ldlr^-/-^* mice, the levels of TG, TC, and LDL-c were significantly reduced in both Alisol A treatment groups (Figure 1B, *P* < 0.05, *P* < 0.01). The total cholesterol levels in the brain tissue of mice were measured, and the results showed that, compared to the control group, the total cholesterol in the brain tissue of *Ldlr^-/-^* mice was significantly increased. In contrast, the brain tissue total cholesterol in the Alisol A treatment group was significantly decreased compared to the *Ldlr^-/-^ mice* (Figure 1C, *P* < 0.01). ORO and HE staining were used to determine the percentage of lipid content in the aortic root plaques (Figure 1D-G). Compared to the control group, *Ldlr^-/-^* mice exhibited larger plaque areas and higher lipid content. In contrast, the plaque area and lipid content in the aortic root of the Alisol A treatment group were significantly reduced compared to the *Ldlr^-/-^* mice (Figure 1D-G, *P* < 0.01). α-smooth muscle actin (α-SMA) is an actin isoform that provides tension and contraction functionality, while CD68 is a marker of macrophages, commonly used to assess plaque inflammation. Compared to the control group, *Ldlr^-/-^* mice exhibited elevated expression of α-SMA and CD68 in atherosclerotic plaques, indicating increased smooth muscle cell activation and macrophage infiltration. However, treatment with Alisol A inhibited the expression of α-SMA and CD68 (Figure 1H-I, *P* < 0.05, *P* < 0.01). In conclusion, Alisol A treatment not only reduced the area of atherosclerotic lesions but also improved the cellular composition within the atherosclerotic plaques. To assess the effects of Alisol A on learning and memory in mice, MWM, Y-maze, and NOR tests were conducted. Alisol A decreased avoidance latency in *Ldlr^-/-^* mice during learning (Figure 1J-K) and increased time in target quadrant (Figure 1L-M). Y-maze test showed reduced spontaneous alternations in *Ldlr^-/-^* mice, indicating impaired spatial working memory. *Ldlr^-/-^* mice showed a significant preference for the new arm and an apparent increase in spontaneous alternation after Alisol A treatment, revealing sustained improved spatial short-term memory (Figure 1N-Q). The control group preferred the new object in the NOR task, but *Ldlr^-/-^* mice did not (Figure 1R-U). Alisol A supplementation prevented the decrease in novel object discrimination in *Ldlr^-/-^* mice. These findings indicate that Alisol A has a beneficial impact on cognitive functions and memory in *Ldlr^-/-^* mice.

### Alisol A protects hippocampus neurons from cholesterol metabolism disorders and oxidative stress damage

In the *in vitro* study, HT22 cells were exposed to Aβ_25-35_ and a cholesterol complex (50 μg/ml cholesterol) 20, and primary neuronal cells isolated from the brains of neonatal mice were co-incubated with ox-LDL to simulate neuronal cholesterol metabolism disorders. Subsequently, the expression of cholesterol metabolism-related genes in the cells was analyzed by Western blot. CYP46A1 catalyzes the conversion of cholesterol to 24S-OHC, facilitating cholesterol's passage across the blood-brain barrier and activating LXR signaling 91. SREBP2 and LXR-α work together to regulate cholesterol uptake, synthesis, and efflux, thereby maintaining intracellular cholesterol homeostasis 92, 93. The activation of SREBP2 is associated with the upregulation of 3-hydroxy-3-methylglutaryl-coenzyme A reductase (HMGCR) 94. Furthermore, LXR promotes cholesterol efflux by activating the transcription of ATP-binding cassette transporter A1 (ABCA1) 95.

**Figure 2:**
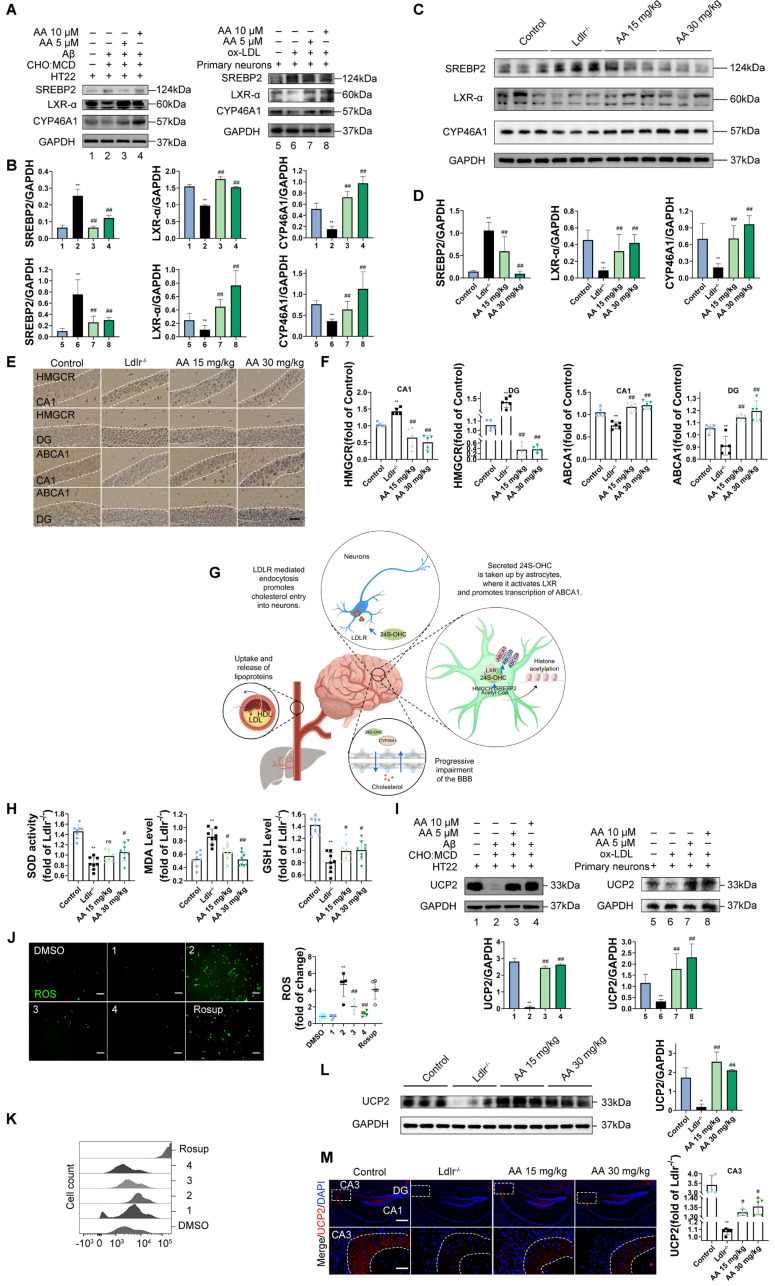
** Alisol A protects hippocampus neurons from cholesterol metabolism disorders and oxidative stress damage. (A)** Key cholesterol metabolism proteins were validated by WB: SREBP2, LXR-α, CYP46A1; and** (B)** WB bands were quantified by Image J, n = 3. **(C)** Total protein was collected from the mice hippocampal tissues and evaluated by WB, including SREBP2, LXR-α, and CYP46A1, bar graph represents semi-quantification of WB **(D)**, n = 3; **(E)** Representative IHC staining images of hippocampal neurons in CA1 and dentate gyrus (DG) zones (counterstaining is blue and positive staining is brown), including HMGCR, and ABCA1, and quantification of IHC **(F)**, scale: 50 μm, n = 5; **(G)** Schematic. **(H)** Respectively, levels of GSH, MDA, and SOD in the hippocampus of mice were determined with GSH, MDA, and SOD assay kits, n = 8; **(I)** WB analysis of UCP2 in HT22 cells, and quantification result was normalized against the levels of GAPDH, n = 3; **(J)** ROS detection was conducted utilizing a DCFH-DA cellular ROS detection assay, scale: 100 μm, n = 5; (**K**) Flow cytometry analysis of HT22 cells was conducted to detect intracellular ROS under various treatment conditions; **(L)** Representative results of WB analysis and quantification results were normalized against the levels of GAPDH, n = 3; **(M)** Representative IF images of hippocampal neurons in CA3 regions, scale: 100 μm, enlarge scale: 10 μm, n = 5; IF was quantified for UCP2 (red). Compared to the 1, ^*^P < 0.05, ^**^P < 0.01; compared to the 2, ^#^* P* < 0.05, ^##^* P* < 0.01. 1: HT22; 2: HT22+ Aβ_25-35_ (20 μM) and complex (50 μg/ml of cholesterol); 3: HT22+ Aβ_25-35_ (20 μM) and complex (50 μg/ml of cholesterol) + Alisol A 5 μM; 4: HT22+ Aβ_25-35_ (20 μM) and complex (50 μg/ml of cholesterol) + Alisol A 10 μM. Rosup: HT22 + Aβ_25-35_ (20 μM) a complex (50 μg/ml of cholesterol) + H_2_O_2_ 0.1mM. Compared to the 5, ^*^ P < 0.05, ^**^ P < 0.01; compared to the 6, ^#^* P* < 0.05, ^##^* P* < 0.01. 5: Primary neurons; 6: Primary neurons+ ox-LDL (70 μg/ml); 7: Primary neurons+ ox-LDL (70 μg/ml) + Alisol A 5 μM; 8: Primary neurons+ ox-LDL (70 μg/ml) + Alisol A 10 μM. Compared to the control group, ^*^ P < 0.05, ^**^ P < 0.01; compared to the *Ldlr^-/-^*, ^#^* P* < 0.05, ^##^* P* < 0.01.

First, in the in vitro cell experiments, the results showed that, compared with the control group, in both HT22 cells treated with Aβ_25-35_ plus cholesterol complex (50 μg/mL cholesterol) and primary mouse neurons treated with ox-LDL, SREBP2 expression was significantly upregulated, while LXR-α and CYP46A1 expression were significantly downregulated (Figure 2A-B, *P* < 0.01). However, following Alisol A treatment, SREBP2 expression was markedly reduced, whereas LXR-α and CYP46A1 expression were significantly increased (Figure 2A-B, *P* < 0.01). In the *in vivo* animal experiments, compared with the control group, *Ldlr^-/-^* mice exhibited decreased LXR-α and CYP46A1 expression and increased SREBP2 expression in hippocampal tissue (Figure 2C-D, *P* < 0.01). After Alisol A treatment, LXR-α and CYP46A1 expression were significantly upregulated, and SREBP2 expression was significantly downregulated (Figure 2C-D, *P* < 0.05, *P* < 0.01). Immunohistochemical analysis showed that, compared with controls, *Ldlr^-/-^* mice had reduced ABCA1 and increased HMGCR staining in the hippocampus; Alisol A treatment reversed these changes, increasing ABCA1 and decreasing HMGCR (Figure 2E-F, *P* < 0.01). These findings suggest that Alisol A may correct neuronal cholesterol overload by inhibiting SREBP2- and HMGCR-mediated cholesterol synthesis while activating LXR-α-, CYP46A1-, and ABCA1-mediated cholesterol efflux and metabolism.

Figure 2G illustrates the mechanistic link between cholesterol metabolism and vascular cognitive impairment. Abnormal cholesterol metabolism, particularly cholesterol accumulation due to LDLR dysfunction, is a significant trigger for neurological disorders 91. Cholesterol metabolism dysregulation serves as a key molecular bridge linking atherosclerosis and cognitive impairment. Elevated total cholesterol and low-density lipoprotein cholesterol in the circulation accelerate atherosclerotic plaque formation, and their metabolic product, oxysterols, can cross the BBB. This, in turn, interferes with brain cholesterol homeostasis by activating LXR signaling. Peripheral ox-LDL not only promotes atherosclerotic plaque development but also crosses the damaged BBB into the brain parenchyma, activating abnormal ABCA1 transport in astrocytes, thereby exacerbating cholesterol deposition in the central nervous system 95. This mechanism deepens the synergistic interaction between cholesterol metabolism dysregulation in the nervous system and neurodegenerative damage in the brain. Systemic disruption of cholesterol metabolism constitutes a critical bridge for "cardio-cerebral comorbidity."

Multiple studies have shown that high cholesterol exacerbates oxidative stress and impairs neuronal function 96, 97. To further investigate the mechanisms by which cholesterol metabolism disorder damages the nervous system, assess the therapeutic effects of Alisol A, and clarify the link between oxidative stress and atherosclerosis related vascular cognitive impairment, we measured oxidative stress related indicators. UCP2 (Uncoupling Protein 2) is a mitochondrial inner-membrane uncoupling protein that is widely expressed in the central nervous system. UCP2 is regarded as a core component of the antioxidant defense system: by dissipating the mitochondrial membrane potential, it inhibits excessive ROS production, thereby alleviating oxidative damage and protecting neuronal survival 98. In the *in vivo* experiments, the results of this study revealed that, compared to the control group, *Ldlr^-/-^* mice exhibited a significant decrease in GSH and SOD activity in the hippocampus, a downregulation of UCP2 expression, and a significant increase in MDA levels. However, Alisol A treatment significantly increased UCP2 expression, as well as significantly improved GSH and SOD activity in the hippocampus of *Ldlr^-/-^* mice, while simultaneously reducing MDA levels (Figure 2H, Figure 2L-M, *P* < 0.05, *P* < 0.01).

In the *in vitro* experiments, DCFH-DA fluorescence was used to label intracellular ROS, and flow cytometry was employed for analysis and quantification. The results showed that, compared to the control group, UCP2 expression was significantly downregulated in HT22 cells treated with Aβ_25-35_ and cholesterol complex (50 μg/ml cholesterol) and in primary mouse neuronal cells treated with ox-LDL. However, following Alisol A treatment, UCP2 expression was significantly upregulated compared to the model group (Figure 2I, *P* < 0.01). Additionally, compared to the control group, ROS levels were significantly elevated in HT22 cells treated with Aβ_25-35_ and cholesterol complex (50 μg/ml cholesterol). After Alisol A treatment, ROS levels were significantly reduced compared to the model group (Figure 2J-K, *P* < 0.05, *P* < 0.01).

### Alisol A exerts neuroprotective effects in *Ldlr^-/-^* mice by attenuating neuroinflammatory impairments and enhancing hippocampal synaptic plasticity

**Figure 3:**
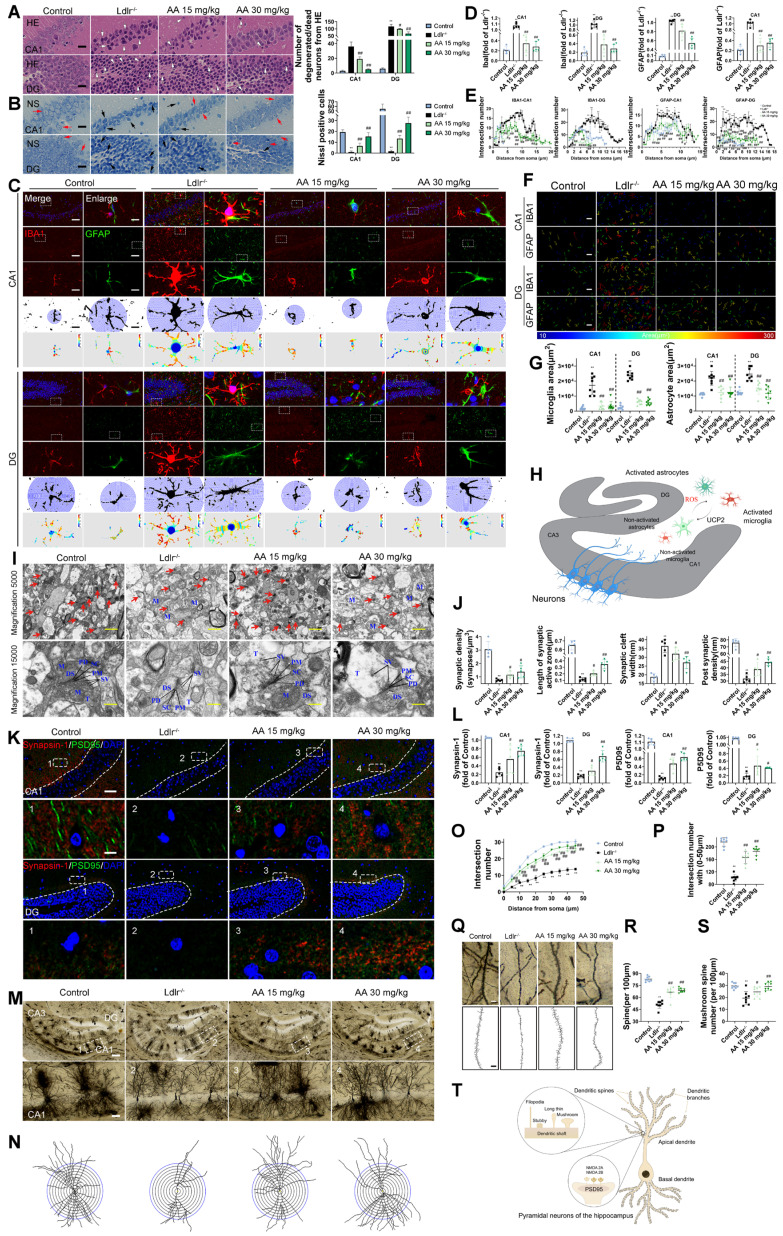
** Alisol A treatment improved neural cell morphology and inhibited the activation of astrocytes and microglia, and restored synaptic loss and impairment of *Ldlr^-/-^* mice. (A)** HE staining shows morphological changes of neurons in the hippocampal CA1 and DG regions, scale: 5 μm, n = 5; White arrows indicate degenerating neurons, characterized by nuclear pyknosis, increased cytoplasmic eosinophilia, and disorganized arrangement. **(B)** Nissl staining reveals the distribution of Nissl bodies, scale: 5 μm, n = 5; Red arrows indicate Nissl-positive neurons, while black arrows denote degenerating neurons. Quantification includes the number of degenerating neurons and Nissl-positive cells. **(C)** Representative immunofluorescence images of hippocampal neurons in the CA1 and DG regions were analyzed. An enlarged view is provided on the right, showcasing representative images of IBA1 ^+^ microglia and GFAP ^+^ astrocytes, accompanied by Sholl analysis. The intersection number per radius over the distance from the cell body was displayed graphically in the curve, scale: 10 μm, enlarge scale: 2 μm;** (D)** IF was quantified for IBA1 (red) and GFAP (green), n = 5; **(E)** Line chart of intersection number per radius versus distance from the cell body; **(F)** Representative reconstruction images of astrocytes and microglia, with the cellular regions delineated using distinct colors, were obtained, scale: 10 μm; **(G)** The areas occupied by astrocytes and microglia were subsequently quantified and subjected to analysis; (**H**) Schematic. **(I)** Alterations in hippocampus ultrastructure were examined by transmission electron microscope (TEM), mitochondria (M), axonal terminal (T), presynaptic density membrane (PM), synaptic vesicle (SV), dendritic spine (DS), postsynaptic density membrane (PD), synaptic cleft (SC), scale: 2 μm, enlarge scale: 1 μm, n = 5; **(J)** A quantitative analysis of the ultrastructural synapses in *Ldlr^-/-^* mice was conducted, focusing on the number of synaptic vesicles (SVs) per synaptic profile, the synaptic length within the active zones, the width of the synaptic cleft, and the thickness of the post-synaptic density; **(K)** Synapses in the hippocampal CA1 and DG region are revealed by IF staining for the pre-and postsynaptic markers, Synapsin-1 (red) and PSD95(green), respectively, scale: 10 μm, enlarge scale: 2 μm, n = 5; **(L)** and quantification of IF; **(M)** Golgi-Cox Staining Hippocampal Golgi-Cox staining was performed as previously described, scale: 100 μm, enlarge scale: 20 μm, n = 8;** (N)** Track the neural branches after Golgi staining;** (O)** and **(P)** analysis of Sholl results and statistical data; **(Q)** and **(R)** analysis of dendritic spine density in neurons stained with Golgi, along with the quantification of mushroom spines, scale: 2 μm** (S)**;** (T)** Schematic. Compared to the control group, ^*^ P < 0.05, ^**^ P < 0.01; compared to the *Ldlr^-/-^* mice, ^#^* P* < 0.05, ^##^* P* < 0.01.

HE and Nissl staining were used to analyze hippocampal neuronal morphology in the CA1 and DG regions. The control group showed normal hippocampal morphology with well-organized cell layers and consistent staining (Figure 3A-B). Conversely, in the *Ldlr^-/-^* mice, the hippocampal cell with some exhibiting necrosis or being absent, and the neuronal nuclei appeared shrunken. The Alisol A 15 mg/kg and Alisol A 30 mg/kg groups had better pyramidal cell numbers and structure in the hippocampus, as well as increased Nissl bodies in neurons compared to the *Ldlr^-/-^* mice.

Subsequently, we examined glial inflammatory responses through immunostaining for the glial markers IBA1 (microglia) and GFAP (astrocytes) (Figure 3C). Immunostaining for IBA1 and GFAP was increased in the hippocampus of *Ldlr^-/-^* mice, indicating glial activation in response to neuronal damage (Figure 3C-D). Following Alisol A treatment, neuroinflammation was significantly reduced, as demonstrated by decreased IBA1 and GFAP staining (Figure 3E). Assess the complexity and structure of microglia and astrocytes using Sholl analysis. Figure 3C illustrates the use of concentric circles to measure their branching. The model group exhibited a significant increase in the number of intersections with the circles. Specifically, the *Ldlr^-/-^* mice demonstrated a significant rise in intersection points per radius and an increased distance to the cell body (Figure 3C), accompanied by an elevated presence of microglia and astrocytes in the hippocampus (Figure 3F-G). This scenario can be elucidated as follows: Amid oxidative stress, the proliferation of ROS stimulates the activation of microglia and astrocytes. In parallel, UCP2, a protein residing in the mitochondrial inner membrane, alleviates oxidative stress and curbs neuroinflammation by moderating ROS levels, which in turn shields the mitochondria from damage (Figure 3H).

Synaptic dysfunction in the hippocampus is the primary cause of cognitive decline 99, 100. The present study employed TEM to investigate synaptic structure in the hippocampus. The *Ldlr^-/-^* mice showed fewer synapses, narrower synaptic clefts, and smaller postsynaptic densities compared to the control group (Figure 3I-J). However, these impairments were ameliorated by Alisol A treatment. Immunofluorescence assay targeting the presynaptic protein synapsin-1 and the postsynaptic protein PSD95 revealed that both proteins were down-regulated in the *Ldlr^-/-^* mice, indicating a loss of synapses. Alisol A treatment enhanced the eservation of presynaptic and postsynaptic proteins in the hippocampi of *Ldlr^-/-^* mice (Figure 3K-L). The hippocampi underwent Golgi-Cox staining in order to observe the dendritic spine structure within neurons (Figure 3M-S). Sholl analysis revealed decreased dendritic complexity and spine density in hippocampal CA1 neurons of *Ldlr^-/-^* mice compared to controls. Notably, neurons in the model group exhibit a reduction in the number of mature spines (mushroom-type), which is accompanied by an overall decrease in spine number. Treatment with Alisol A resulted in significant improvements in these parameters. Specifically, the groups administered Alisol A at dosages of 15 mg/kg and 30 mg/kg exhibited increased dendritic branching, enhanced spine density, and a higher number of mushroom spines in comparison to the model group. The increase in mushroom-shaped spines carries dual significance. On one hand, their enlarged heads markedly expand the synaptic membrane contact area, thereby enhancing the stability of synaptic connections. On the other hand, such spines are typically indicative of mature synapses, which exhibit higher functional activity and greater efficiency in information transmission. Therefore, Alisol A may exert more targeted neuroprotective and cognitive-enhancing effects by reinforcing structural characteristics of mature synapses, rather than merely promoting dendritic branching complexity (Figure 3T).

### AMPK pathway and autophagy may be key mechanisms of Alisol A, with NAMPT playing an important role in their regulation

**Figure 4:**
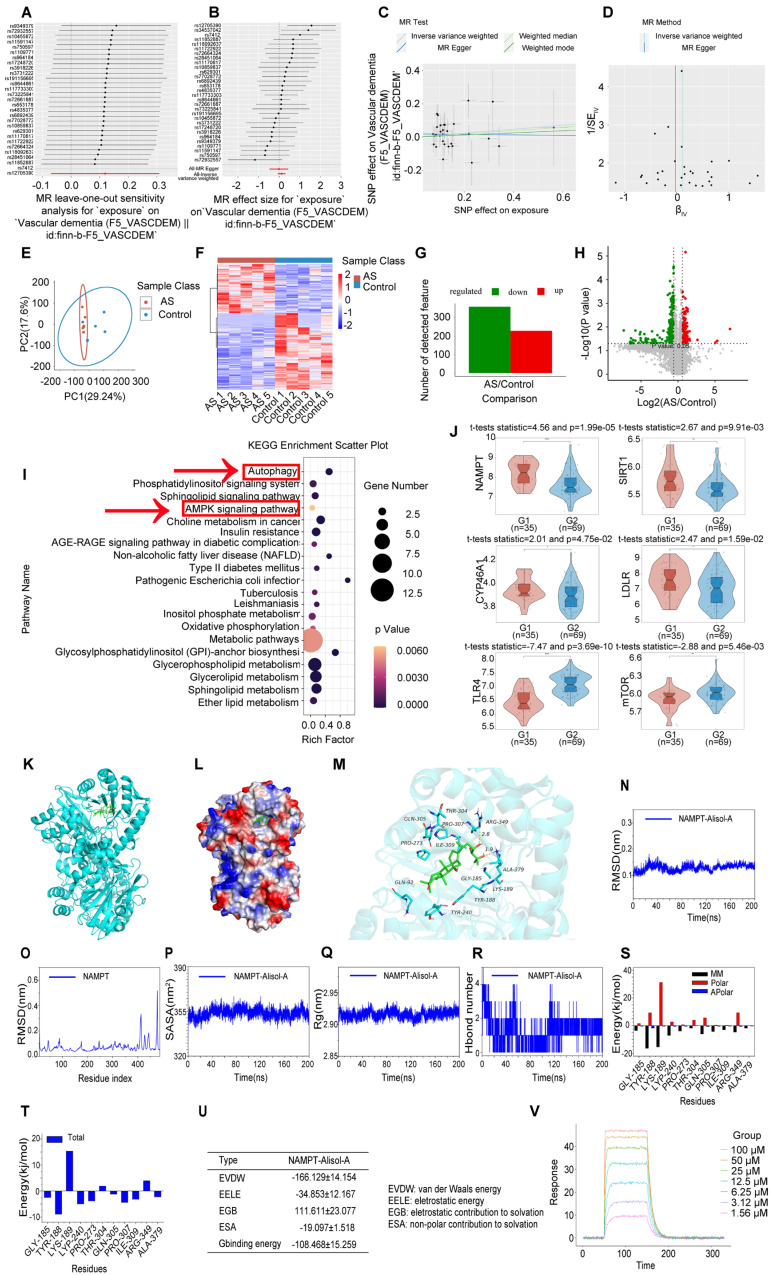
** Atherosclerosis has the potential to impact cognitive function via the autophagy and AMPK signaling pathways, with Alisol A capable of binding to NAMPT and modulating its activity. (A-D)** Using Mendelian randomization to explore the causal link between coronary atherosclerosis and vascular dementia. **(A)** A leave-one-out sensitivity analysis was performed to assess if any single SNP disproportionately influences the association. In the forest plot, each black point shows the Mendelian Randomization analysis results excluding that specific SNP. **(B)** The causal effect of exposure on the outcome is evaluated for each SNP using the Wald ratio and shown in a forest plot. The overall Mendelian Randomization (MR) estimate, using all SNPs and various MR methods, is also provided. **(C)** The effects of SNPs on the outcome are plotted against their effects on the exposure, with each line's slope indicating causal association. Each method produces a unique line, except for the Egger estimate, which is the only line not passing through the origin. **(D)** A funnel plot was used to assess heterogeneity, with less precise estimates forming a funnel shape as precision increases. A wider spread indicates more heterogeneity, possibly due to horizontal pleiotropy. Asymmetry implies directional pleiotropy that biases many MR methods, but MR Egger regression can reduce this bias. **(E-I)** Serum Lipidomics, n = 5; **(E)** Scatter plot of Principal Component Analysis (PCA) scores; **(F)** Heatmap illustrating differential metabolites in the comparison group; **(G)** Bar chart depicting differential statistics of metabolic ions; **(H)** Diagram illustrating the differential metabolites in volcanic activity; **(I)** Bubble Chart of differential metabolite enrichment pathways in the KEGG database;** (J)** Analysis of gene expression profiles in the GEO dataset (ID: GSE100927, G1 = 35: Standard control participants, G2 = 69: Patients with atherosclerosis). The binding mode of NAMPT with Alisol A after molecular dynamics, **(K)** the 3D structure of complex; **(L)** the Electrostatic surface of protein; **(M)** The detail binding mode between Alisol A and NAMPT; The backbone of protein was rendered in a tube and colored in cyan; Compound is rendering by red, Yellow dash represents hydrogen bond distance; The RMSD of Alisol A with NAMPT **(N)**; **(O)** The RMSD of Alisol A with NAMPT; **(P)** The SASA of Alisol A with NAMPT; **(Q)** The R go f Alisol A with NAMPT; **(R)** The hydrogen bond number of Alisol A with NAMPT; **(S) (T)** The energy decomposition for the interaction of Alisol A with NAMPT; molecular mechanics (MM), polar solvation energy (Polar), and nonpolar solvation energy (Apolar); **(U)** The binding energy by MMG-NAMPT (kJ/mol);** (V)** SPR, Sensorgrams for the interaction of NAMPT and Alisol A.

We conducted a two-sample mendelian randomization study, utilizing data from GWAS, to elucidate the causal effect of coronary atherosclerosis on vascular dementia (Figure 4A-D). Heterogeneity and pleiotropy tests indicate no significant heterogeneity or pleiotropy in this study, suggesting that the Mendelian randomization analysis is reliable (Table S1 and S2). When the odds ratio (OR) is greater than 1, it indicates that coronary atherosclerosis is a risk factor for vascular dementia (i.e., it increases the risk of disease). In this study, the OR values were as follows: IVW: OR = 1.238 (*P* < 0.05); MR Egger: OR = 1.238; Weighted median: OR = 1.238 (Table S3). To investigate the key enrichment pathways of differential metabolites between atherosclerosis patients and a normal blood lipid control group, we conducted lipidomics analyses on the serum of both atherosclerosis patients and the control group (Figure 4E-I). KEGG pathway enrichment analysis (Figure 4I) revealed that the differentially expressed metabolites were primarily concentrated in key metabolic pathways such as the AMPK signaling pathway and autophagy pathway. In the bubble plot, pathways with a larger Rich factor and smaller P-value indicated higher enrichment levels, suggesting that these metabolic pathways may play an important role in the mechanism of Atherosclerosis related vascular cognitive impairment. The AMPK signaling pathway and autophagy pathway may be key mechanisms through which Alisol A exerts its effects in atherosclerosis-related diseases. Next, the study analyzed publicly available atherosclerosis datasets from GEO (GSE100927). GEO dataset analysis revealed that the expression of *NAMPT, SIRT1, CYP46A1*, and *LDLR* was significantly reduced in atherosclerosis patients (*P* < 0.05 or *P* < 0.01), while *TLR4* and *mTOR* levels were significantly increased (Figure 4J, *P* < 0.05 or *P* < 0.01). Among these, the difference in *NAMPT* expression was most significant, suggesting that *NAMPT* may play an important role in the regulation of the AMPK pathway and autophagy.

Molecular docking simulation demonstrated Alisol A binding to NAMPT, forming hydrogen bonds at GLN-305 and TYR-188 residues. This suggests Alisol A could be an effective agonist for NAMPT activity Figure 4K-M. The results obtained from molecular docking analyses were subsequently confirmed through an additional 200 ns of MD. It can be seen in the RMSD protein graphs (Figure 4N-O) that average RMSD values are less than 2 Å and the complex reached a dynamic equilibrium at around 80 ns. This finding evinces that Alisol A peptides interacted and formed stable molecular complexes with NAMPT protein. Notably, as illustrated in Figure 4P-R the protein-ligand complex exhibited minimal fluctuations throughout the entire simulation period. This observation suggests that the compound remained within the protein pocket, thereby demonstrating a stable binding affinity conducive to achieving dynamic equilibrium. In addition to these, hydrogen bonding interactions of Alisol A with critical residues in the active site of ARG-349, and ALA-379. Alisol A could also form hydrophobic interactions with multiple amino acid residues such as PRO-307, TYR-240, TYR-188, ALA-379, PRO-273, and ILE-309. In Figure 4S and Figure 4T, the main forces between the Alisol A protein and the NAMPT complex were MM, Polar, and Apolar energies. And GLY-185, TYR-188, TYR-240, PRO-273, GLN-305, PRO-307, ILE-309, and ALA-379 with a higher contribution for the Alisol A protein and NAMPT complex. Based on the data presented in Figure 4U, it can be observed that the van der Waals force exhibits a superior ability to stabilize small molecules, with electrostatic interactions ranking second in effectiveness. The study found that Alisol A binds to the NAMPT protein with a binding free energy of -108.468 ± 15.259 kJ/mol, primarily due to van der Waals force (-166.129 ± 14.154 kJ/mol). Overall, Alisol A has exhibited a strong binding affinity for NAMPT. NAMPT immobilized on CM5 chip can bind Alisol A with an affinity constant of 65.1 μM as determined in a SPR assay (Figure 4V). These binding affinities enhance the interaction between Alisol A and NAMPT, resulting in the creation of a stable "high affinity" complex that facilitates the exertion of its functional role.

### Alisol A activates the AMPK/NAMPT/SIRT1 pathway and autophagy pathway

Peroxisome proliferator-activated receptor gamma coactivator 1-alpha (PGC1-α) is a key cofactor of SIRT1 and plays a role in mitochondrial function and biogenesis 101. SIRT1, an NAD⁺-dependent deacetylase, promotes the initiation and maintenance of autophagy by deacetylating key autophagy proteins such as Atg5, Atg7, and Atg8 102, 103. NAMPT, by regulating intracellular NAD⁺ levels, indirectly activates SIRT1 and regulates the autophagic process. Measuring lysine acetylation levels helps assess how SIRT1 and NAMPT regulate the activity of autophagic factors through acetylation.

**Figure 5:**
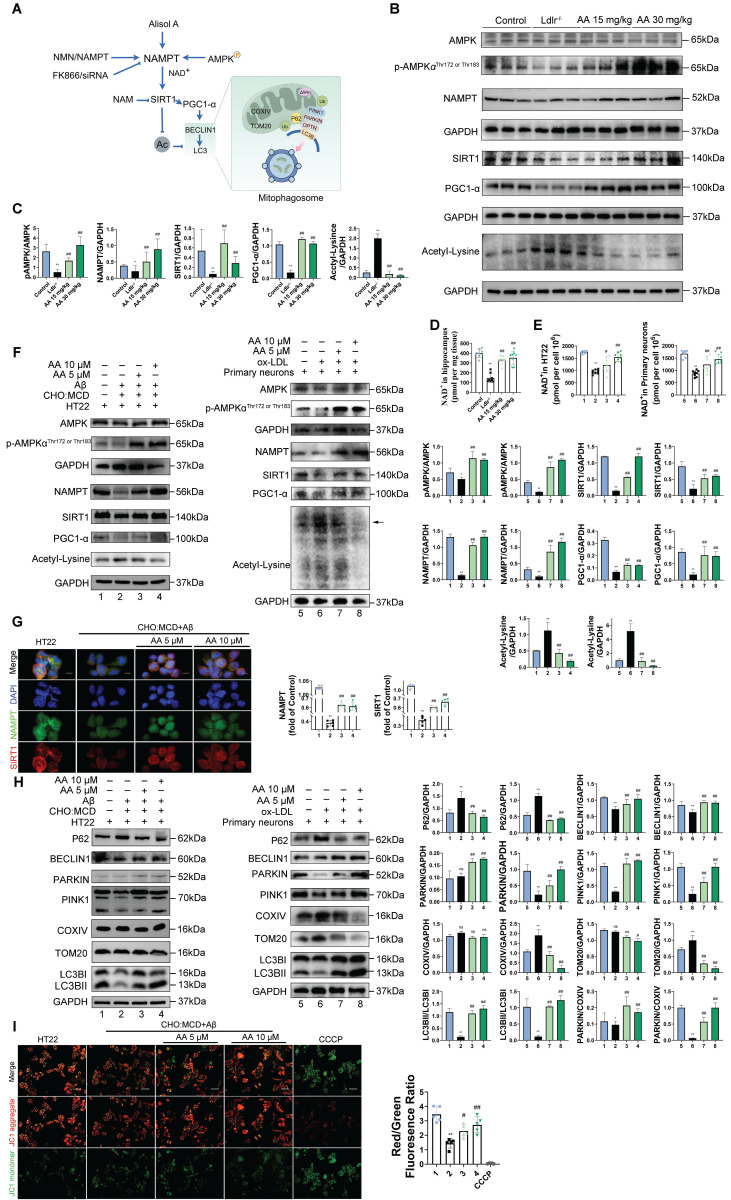
** Alisol A activates the NAMPT-related signaling pathway, thereby facilitating lysine deacetylation and autophagy. (A)** Schematic. **(B)** Total proteins were collected from the mice hippocampal tissues and evaluated by WB, including AMPK, p-AMPKα ^Thr172 or Thr183^, NAMPT, SIRT1, PGC1-α, and AcetyI-Lysine, bar graph represents semi-quantification of WB **(C)**, n = 3;** (D)** NAD^+^ levels in the hippocampus of *Ldlr^-/-^* mice were determined by the NAD^+^ Assay Kit, n = 8;** (E)** NAD⁺ content in HT22 cells or in primary neurons was measured using an NAD assay kit, n = 8; **(F)** WB analysis of AMPK, p-AMPKα ^Thr172 or Thr183^, NAMPT, SIRT1, PGC1-α, and AcetyI-Lysine in cells, and quantification result was normalized against the levels of GAPDH, n = 3;** (G)** Representative images of NAMPT (green) and SIRT1 (red) double immunofluorescence staining, scale: 5 μm; and quantification of immunofluorescence stain intensity using Image J; **(H)** WB analysis of P62, BECLIN1, PARKIN, PINK1, COXIV, TOM20, and LC3B in cells, and quantification result was normalized against the levels of GAPDH or COXIV, n = 3; **(I)** Changes in MMP was monitored by staining with JC1, scale: 100 μm; and quantification of the percentage of MMP, n = 8. Compared to the 5, ^*^ P < 0.05, ^**^ P < 0.01; compared to the 6, ^#^* P* < 0.05, ^##^* P* < 0.01. Compared to the control group, ^*^ P < 0.05, ^**^ P < 0.01; compared to the *Ldlr^-/-^*, ^#^* P* < 0.05, ^##^* P* < 0.01.

Figure 5A presents a schematic diagram illustrating the mechanism by which Alisol A sustains mitochondrial homeostasis through the activation of the AMPK/NAMPT/SIRT1 signaling pathway and the induction of autophagy. In the *in vivo* experiments, this study explored the effects of Alisol A on the hippocampal AMPK/NAMPT/SIRT1 signaling pathway. The results showed that, compared to the control group, *Ldlr^-/-^* mice exhibited significantly decreased levels of p-AMPKα^Thr172 or Thr183^, NAMPT, SIRT1, and PGC1-α proteins, while lysine acetylation (Acetyl-Lysine) was significantly increased (Figure 5B-C, *P* < 0.05, *P* < 0.01). Additionally, NAD^+^ levels were reduced in *Ldlr^-/-^* mice (Figure 5D, *P* < 0.01). However, compared to the *Ldlr^-/-^* group, Alisol A treatment significantly increased the expression of p-AMPKα^Thr172 or Thr183^, NAMPT, SIRT1, and PGC1-α, while lysine acetylation (Acetyl-Lysine) significantly decreased, and NAD^+^ levels were significantly elevated (Figure 5B-D, *P* < 0.05, *P* < 0.01). To further validate that Alisol A activates the AMPK/NAMPT/SIRT1 signaling pathway, we also conducted relevant experiments at the cellular level. The results showed that, compared to the control group, the model group exhibited significantly reduced levels of p-AMPKα^Thr172 or Thr183^, NAMPT, SIRT1, and PGC1-α proteins, while total lysine acetylation (Acetyl-Lysine) was significantly increased. However, compared to the model group, the Alisol A treatment group showed increased expression of p-AMPKα^Thr172 or Thr183^, NAMPT, SIRT1, and PGC1-α, along with a significant decrease in total lysine acetylation (Acetyl-Lysine) (Figure 5F, *P* < 0.05, *P* < 0.01). Additionally, NAD^+^ levels were significantly decreased in the model group compared to the control group, but Alisol A treatment improved this result (Figure 5E, *P* < 0.05, *P* < 0.01). These results suggest that Alisol A activates the AMPK/NAMPT/SIRT1 signaling pathway. IF results also supported the findings (Figure 5G).

To investigate the effect of Alisol A on neuronal mitophagy, we measured the expression of autophagy-related proteins by Western blot. Specifically, we probed the autophagic substrate P62 (Sequestosome 1), the key initiation protein BECLIN1, the mitophagy markers PINK1 and PARKIN, and the mitochondrial membrane proteins TOM20 (Translocase of the outer membrane 20) and COXIV. We also assessed LC3B I (Microtubule-associated protein 1A/1B-light chain 3B I) -to-LC3BII conversion to gauge autophagosome formation, and calculated LC3BII/LC3BI and PARKIN/COXIV ratios as quantitative indices of autophagic flux and mitophagy activity. Mitophagy is essential for removing damaged mitochondria and maintaining mitochondrial quality. P62 is a selective autophagy substrate whose levels inversely correlate with overall autophagic activity; its accumulation indicates impaired autophagy 104, 105. PARKIN, an E3 ubiquitin ligase, is activated and recruited to the outer mitochondrial membrane by PINK1 upon mitochondrial damage or depolarization, thereby initiating mitophagy. PARKIN recruitment marks damaged mitochondria for ubiquitination of their membrane proteins, promoting their enclosure by autophagosomes and subsequent degradation. Cytochrome c oxidase subunit IV (COXIV), a component of respiratory chain complex IV, serves as a mitochondrial inner membrane marker whose levels reflect mitochondrial content. During active mitophagy, clearance of damaged mitochondria leads to decreased COXIV. Therefore, the PARKIN/COXIV ratio measured by Western blot provides a dynamic readout of mitophagy targeting efficiency: an elevated ratio indicates enhanced PARKIN recruitment and mitophagy activity, while a reduced ratio suggests impaired PARKIN mobilization or ubiquitin-mediated mitochondrial clearance.

The results of this study show that, compared to the control group, HT22 cells treated with Aβ_25-35_ and cholesterol complex (50 μg/ml cholesterol), as well as primary mouse neuronal cells treated with ox-LDL, exhibited reduced levels of BECLIN1 and PINK1, elevated levels of P62, and decreased levels of PINK1 and PARKIN. Additionally, in HT22 cells treated with Aβ_25-35_ and a cholesterol complex, the PARKIN/COXIV and LC3II/LC3I ratios were decreased (Figure 5H, *P* < 0.05, *P* < 0.01). Although the expression levels of COXIV and TOM20 were not significantly altered in these cells, the reduction in mitophagy-related protein ratios still suggests impaired autophagic flux. Similarly, in primary mouse neuronal cells treated with ox-LDL, COXIV and TOM20 expression were increased, while the PARKIN/COXIV and LC3II/LC3I ratios were significantly decreased (Figure 5H, *P* < 0.05, *P* < 0.01). These findings indicate that autophagy is inhibited in both HT22 cells exposed to Aβ_25-35_ and cholesterol, as well as in ox-LDL-treated primary neurons. However, Alisol A treatment reversed these changes. In HT22 cells, Alisol A increased BECLIN1 and PINK1 expression, reduced P62 levels, upregulated PARKIN expression, and restored the PARKIN/COXIV and LC3II/LC3I ratios (Figure 5H, *P* < 0.05, *P* < 0.01). Likewise, in primary neurons treated with ox-LDL, Alisol A elevated BECLIN1 and PINK1 expression, decreased P62, enhanced PARKIN expression, reduced COXIV and TOM20 levels, and significantly increased the PARKIN/COXIV and LC3II/LC3I ratios. These results suggest that Alisol A activates the PINK1/PARKIN pathway and restores autophagic activity.

The study indicates that mitochondrial membrane potential (MMP) depolarization is positively correlated with the induction of mitophagy 106-108. MMP depolarization refers to the process of a reduced mitochondrial membrane potential, which can be measured by changes in the red/green fluorescence ratio of JC-1 dye 106, 107. CCCP (carbonyl cyanide m-chlorophenyl hydrazone) is a classic mitochondrial uncoupler that causes MMP depolarization and accelerates cell death, and in this study, it was used as a control drug to induce mitochondrial-related apoptosis 109. The results showed that under CCCP treatment, the JC-1 dye no longer accumulated in the mitochondria, instead existing as green fluorescence, leading to a significant decrease in red fluorescence and an increase in green fluorescence, thus lowering the red/green ratio. This suggests that mitochondrial function was impaired in HT22 cells under CCCP treatment, leading to accelerated cell apoptosis (Figure 5I, *P* < 0.05, *P* < 0.01). Compared to the control group, HT22 cells treated with Aβ_25-35_ and cholesterol complex (50 μg/ml cholesterol) exhibited significantly reduced red fluorescence, increased green fluorescence, and a significantly decreased red/green ratio, indicating MMP depolarization. This further suggests mitochondrial dysfunction and impaired autophagy. In contrast, compared to the Aβ_25-35_ and cholesterol complex (50 μg/ml cholesterol) treated HT22 cells, the 10 μM Alisol A treatment group showed enhanced red fluorescence, increased green fluorescence, and an increased red/green ratio (Figure 5I, *P* < 0.05, *P* < 0.01). These results suggest that Alisol A 10 μM treatment may improve mitochondrial function by restoring MMP.

### NAMPT as a key target in Alisol A's treatment of atherosclerosis related vascular cognitive impairment

To further confirm the protective role of Alisol A is associated with NAMPT, this study used the selective NAMPT inhibitor FK866 to block the NAMPT-related pathway activated by Alisol A. In the *in vivo* experiments, the control group was given NMN, the enzymatic product of NAMPT. In the *in vitro* experiments, NAMPT overexpression was achieved in HT22 cells using a NAMPT lentivirus. Based on previous studies, a dose of 15 mg/kg Alisol A was determined to be optimal for *in vivo* experiments, while 10 μM Alisol A was optimal for *in vitro* experiments. These doses were subsequently used for further experiments.

**Figure 6:**
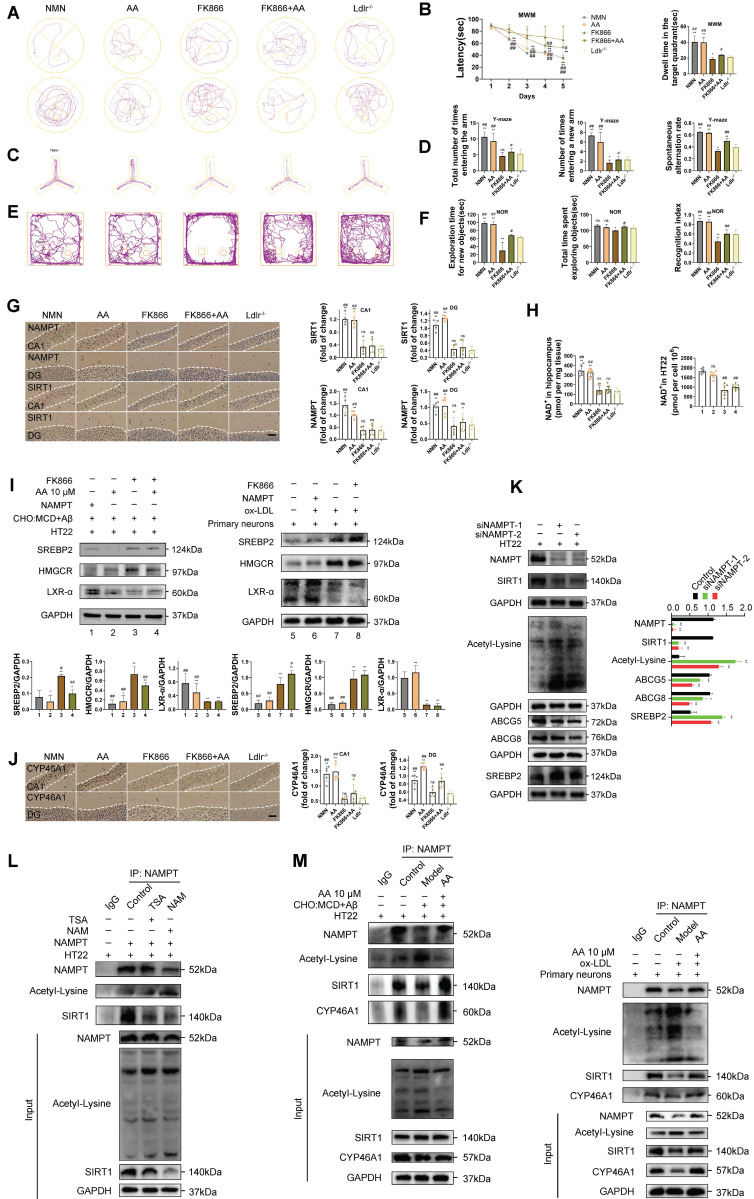
** Inhibiting NAMPT causes cholesterol buildup in the hippocampus, worsening cognitive issues due to reduced NAMPT-SIRT1 interaction, inhibited NAMPT deacetylation, and decreased NAMPT-CYP46A1 interaction. (A-B)** The MWM, the escape latency of the water maze and the mean time to stay in the target quadrant at the last experiment, n = 8; **(C-D)** Y maze, the number of total arm entries, number of entries into the open arm, the spontaneous alternation rate, n = 8; **(E-F)** This conclusion is supported by data from the NOR test, which measured total object exploration time, the duration spent exploring the novel object, n = 8. **(G)** IHC staining of NAMPT and SIRT1 in *Ldlr^-/-^* mice hippocampal tissues in CA1 and DG zones (counterstaining is blue and positive staining is brown) and IHC semi-quantitative of NAMPT and SIRT1, scale: 50 μm, n = 5; **(H)** NAD⁺ levels in the hippocampus of *Ldlr^-/-^* mice and HT22 cells, as determined by the NAD⁺ Assay Kit (n = 8 ); **(I)** WB analysis of SREBP2, HMGCR, and LXR-α in HT22 cells, and quantification result was normalized against the levels of GAPDH, n = 3; **(J)** Representative IHC images of hippocampal neurons in CA1 and DG zones (counterstaining is blue and positive staining is brown), and quantification of IHC, scale: 50 μm, n = 5. **(K)** WB analysis of SIRT1, AcetyI-Lysine, ABCG5, ABCG8, and SREBP2 in HT22 cells, and quantification result was normalized against the levels of GAPDH, n = 3; **(L)** After overexpressing NAMPT in HT22 cells for 48 h, the cells were treated with 1 μM TSA or 5 mM NAM for 12 h. COIP assays with NAMPT antibodies assessed NAMPT acetylation via WB, and its interaction with SIRT1 was checked using SIRT1 antibodies, with IgG as a control;** (M)** HT22 cells were treated with Alisol A (10 μM) or a complex of Aβ_25-35_ (20 μM) and complex (50 μg/ml of cholesterol). Primary neurons were treated with ox-LDL (70 μg/ml). COIP was performed using anti-NAMPT antibodies to assess NAMPT acetylation and its interaction with SIRT1 and CYP46A1, followed by immunoblotting with anti-acetyl-lysine, anti-SIRT1, and anti-CYP46A1 antibodies. IgG served as a negative control. Compared to the *Ldlr^-/-^ mice*, ^*^ P < 0.05, ^**^ P < 0.01; compared to the FK866 group, ^#^* P* < 0.05, ^##^* P* < 0.01. NMN: *Ldlr^-/-^* mice were fed with HFD and treated with NMN 100 mg/kg; Alisol A: *Ldlr^-/-^* mice were fed with HFD and treated with Alisol A 15 mg/kg; FK866: *Ldlr^-/-^* mice were fed with HFD and treated with FK866 25 mg/kg; FK866+ Alisol A: *Ldlr^-/-^* mice were fed with HFD and treated with Alisol A 15 mg/kg and FK866 25 mg/kg. *Ldlr^-/-^*: *Ldlr^-/-^* mice were fed with HFD for the entire study. Compared to the 1, ^*^ P < 0.05, ^**^ P < 0.01; compared to the 3, ^#^* P* < 0.05, ^##^* P* < 0.01. 1:HT22 + Aβ_25-35_ (20 μM) and complex (50 μg/ml of cholesterol) + NAMPT; 2:HT22 + Aβ_25-35_ (20 μM) and complex (50 μg/ml of cholesterol) + Alisol A 10 μM; 3:HT22 + Aβ_25-35_ (20 μM) and complex (50 μg/ml of cholesterol) + FK866 100 nM; 4:HT22 + Aβ_25-35_ (20 μM) and complex (50 μg/ml of cholesterol) + Alisol A 10 μM + FK866 100 nM. Compared to the 5, ^*^ P < 0.05, ^**^ P < 0.01; compared to the 7, ^#^* P* < 0.05, ^##^* P* < 0.01. 5: Primary neurons; 6: Primary neurons+ ox-LDL (70 μg/ml) + NAMPT; 7: Primary neurons+ ox-LDL (70 μg/ml); 8: Primary neurons+ ox-LDL (70 μg/ml) + FK866 100 nM.

The water maze experiment showed that, compared to the *Ldlr^-/-^* group, the FK866 group spent significantly less time in the target quadrant. The NMN and Alisol A treatment groups had a significantly shortened escape latency and spent significantly more time in the target quadrant. Compared to the FK866 group, the NMN and Alisol A treatment groups had a significantly shorter escape latency and significantly more time spent in the target quadrant. The FK866 and Alisol A co-treatment group also showed significantly reduced escape latency and increased time spent in the target quadrant compared to the FK866 group (Figure 6A-B, *P* < 0.05, *P* < 0.01). The Y-maze experiment showed that, compared to the *Ldlr^-/-^* group, the FK866 group had a significantly reduced spontaneous alternation rate. Compared to the FK866 group, the NMN and Alisol A treatment groups had significantly increased entries into the new open arm and a significantly increased spontaneous alternation rate (Figure 6C-D, *P* < 0.05, *P* < 0.01). The novel object recognition experiment showed that, compared to the *Ldlr^-/-^* group, the FK866 group had a significantly reduced novel object discrimination index, while the NMN and Alisol A treatment groups had a significantly increased discrimination index (Figure 6E-F, *P* < 0.05, *P* < 0.01). Immunohistochemistry results showed that FK866 inhibited the expression of NAMPT and SIRT1 in the hippocampus of *Ldlr^-/-^* mice (Figure 6G, *P* < 0.01). Additionally, FK866 significantly reduced NAD^+^ levels (Figure 6H, *P* < 0.01). These findings suggest that NAMPT inhibition exacerbates the already impaired cognitive function and memory in *Ldlr^-/-^* mice.

To investigate the effects of NAMPT on cholesterol metabolism, we examined how modulation of NAMPT expression alters cholesterol homeostasis in both *in vitro* and *in vivo* experiments. Similar to ABCA1, ABCG5 and ABCG8 are regulated by LXR; under conditions of cholesterol overload, LXR is activated by oxysterols 110. Once activated, LXR upregulates the expression of these transporters, thereby promoting cholesterol efflux and reducing intracellular cholesterol levels. After FK866 treatment, we observed increased expression of the key cholesterol-homeostasis regulators SREBP2 and the rate-limiting cholesterol-synthesizing enzyme HMGCR, while LXRα expression was decreased, indicating enhanced cholesterol synthesis and reduced efflux (Figure 6I, *P* < 0.05, *P* < 0.01). In contrast, NAMPT overexpression and treatment with Alisol A produced the opposite effects. Knockdown of NAMPT significantly reduced SIRT1 expression and led to increased lysine acetylation; moreover, NAMPT depletion suppressed the expression of the cholesterol-transport proteins ABCG5 and ABCG8, while upregulating SREBP2 (Figure 6K, *P* < 0.05, *P* < 0.011). Immunohistochemical analysis of mouse hippocampal tissue revealed that FK866 treatment decreased CYP46A1 expression, whereas combined FK866 and Alisol A treatment increased CYP46A1 levels; similarly, NMN or Alisol A alone each significantly elevated CYP46A1 expression (Figure 6J, *P* < 0.05, *P* < 0.01). These findings suggest that inhibition of NAMPT disrupts cholesterol metabolism in neuronal cells.

Protein acetylation is co-regulated by deacetylases (HDACs) and acetyltransferases (HATs). Consequently, we employed two categories of deacetylase inhibitors to target distinct deacetylase activities: type I and type II HDAC family inhibitors, specifically TSA, and a type III (Sirtuins family) inhibitor, NAM. To ensure robust participation of NAMPT in immunoprecipitation assays, we used lentiviral overexpression to substantially increase NAMPT levels. This ensured sufficient protein abundance for detection of its interactions with other proteins. Compared with the IgG control, SIRT1, CYP46A1, and acetyl-lysine modified proteins were all detected in NAMPT immunoprecipitates, indicating that NAMPT interacts with SIRT1, CYP46A1, and acetylated lysine residues and is involved in protein acetylation (Figure 6L-M, *P* < 0.05, *P* < 0.01).

Relative to the non-IgG control, both TSA- and NAM-treated groups exhibited markedly reduced SIRT1 co-precipitation and significantly increased lysine acetylation. Moreover, TSA treatment produced a significantly lower acetyl-lysine signal than NAM treatment, indicating that NAMPT-mediated protein acetylation is primarily regulated by Sirtuin family deacetylases, particularly SIRT1 (Figure 6L, *P* < 0.05, *P* < 0.01). In HT22 cells treated with Aβ_25-35_ plus cholesterol complex and in primary neurons treated with ox-LDL, SIRT1 and CYP46A1 co-immunoprecipitation with NAMPT was significantly reduced, while acetyl-lysine levels were significantly elevated compared with the non-IgG control. These results suggest that Aβ_25-35_+cholesterol and ox-LDL impair the interaction between NAMPT, SIRT1, and CYP46A1 and promote protein acetylation. Finally, in primary neurons treated with Aβ_25-35_+cholesterol or ox-LDL, co-treatment with Alisol A enhanced the interaction between NAMPT, SIRT1, and CYP46A1 and significantly suppressed lysine acetylation (Figure 6M, *P* < 0.05, *P* < 0.01).

### The inhibition of Nampt attenuates autophagy by diminishing its interaction with PINK1, consequently decreasing the advantageous effects of Alisol A in mitigating oxidative stress and restoring synaptic plasticity

**Figure 7:**
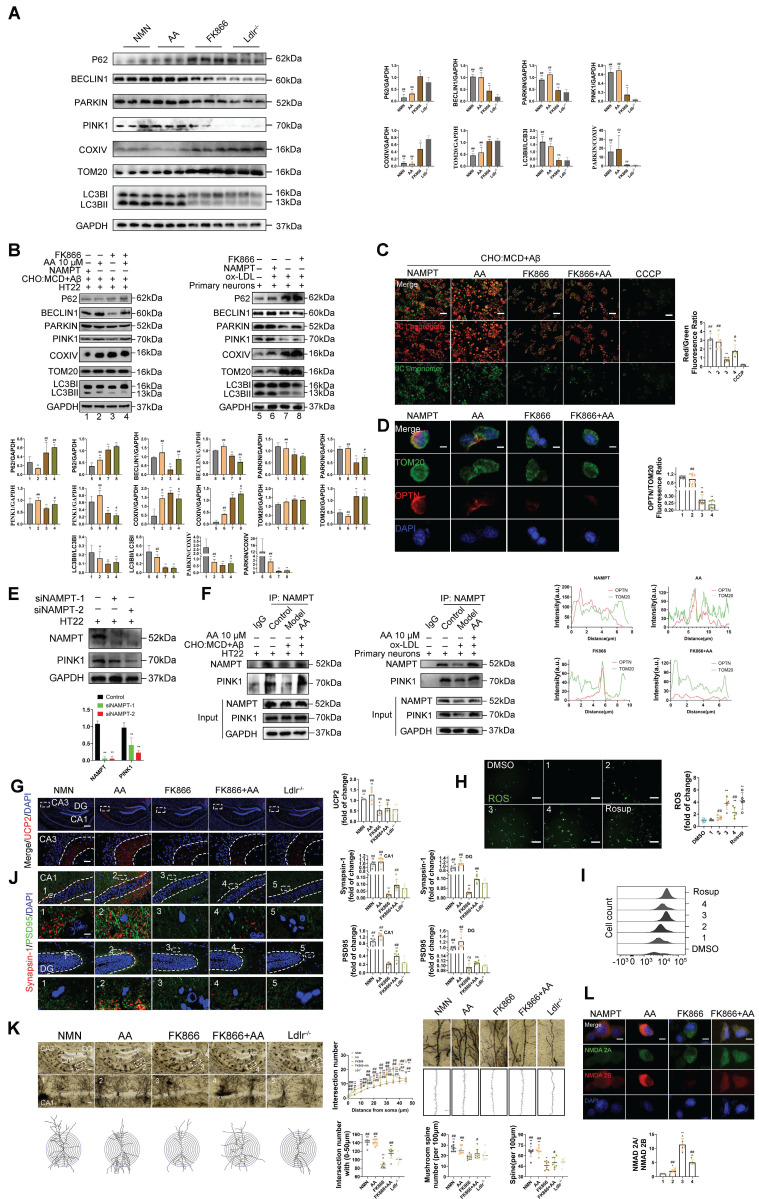
** Modulation of NAMPT activity and its network by Alisol A restores cholesterol balance, mitophagy, and synaptic function in *Ldlr^-/-^* mice. (A)** WB analysis of P62, BECLIN1, PARKIN, PINK1, COXIV, TOM20, and LC3B protein quantitation for WB, n = 3. **(B)** WB analysis of P62, BECLIN1, PARKIN, PINK1, COXIV, TOM20, and LC3B in cells, and quantification result was normalized against the levels of GAPDH or COXIV, n = 3; **(C)** Changes in MMP was monitored by staining with JC1 in HT22 cells, scale: 100 μm; The ratio of red/green fluorescence intenity was employed to assess MMP, n = 8; **(D)** Representative IF images of OPTN and TOM20 in HT22 cells, scale: 2 μm; Quantification of IF stain intensity using Image J, n = 8; IF and colocalization analysis. **(E)** WB analysis of PINK1 in HT22 cells and quantification result was normalized against the levels of GAPDH, n = 3; **(F)** Subsequent COIP experiments employed NAMPT antibodies to assess interactions with NAMPT and PINK1, using IgG as a control. **(G)** Illustrative IF images depicting hippocampal neurons located within the CA3 subregion, scale: 100 μm, enlarge scale: 10 μm, n = 5; IF was quantified for UCP2 (red); **(H)** The phenomenon of intercellular ROS generation was observed through the detection of intracellular ROS levels using the DCFH-DA probe following different treatment protocols, scale: 100 μm, n = 5; **(I)** Intracellular ROS levels were measured using flow cytometry, comparative analysis of fluorescence intensity within peak plots derived from flow cytometry, n = 5; **(J)** Synapses in the hippocampal CA1 and DG region were revealed by IF staining for the pre-and postsynaptic markers, Synapsin-1 (red) and PSD95 (green) respectively, scale: 10 μm, enlarge scale: 2 μm, n = 5; and quantification of IF; **(K)** Golgi-Cox Staining Hippocampal Golgi-Cox staining was performed as previously described, scale: 100 μm, enlarge scale: 20 μm, n = 8; Track the neural branches after Golgi staining and analysis of Sholl results and statistical data; Analysis of dendritic spine density in neurons stained with Golgi, along with the quantification of mushroom spines, scale: 2 μm; **(L)** Representative IF images of NMDA 2A (green) and NMDA 2B (red), scale: 2 μm; and quantification of IF, n = 5. Compared to the *Ldlr^-/-^ mice*, ^*^ P < 0.05, ^**^ P < 0.01; compared to the FK866 group, ^#^* P* < 0.05, ^##^* P* < 0.01. Compared to the 1, ^*^ P < 0.05, ^**^ P < 0.01; compared to the 3, ^#^* P* < 0.05, ^##^* P* < 0.01. DMSO: HT22 +DMSO; Rosup: HT22 + Aβ_25-35_ (20 μM) and complex (50 μg/ml of cholesterol) +H_2_O_2_ 0.1 mM_;_ CCCP: HT22+ CCCP 10 μM. Compared to the 5, ^*^ P < 0.05, ^**^ P < 0.01; compared to the 7, ^#^* P* < 0.05, ^##^* P* < 0.01.

To investigate the role of NAMPT in regulating mitophagy, we performed various interventions in *Ldlr^-/-^* mice and assessed the expression of multiple autophagy related proteins in hippocampal tissue by Western blot. Compared with the *Ldlr^-/-^* group, mice treated with Alisol A or NMN exhibited significantly increased levels of BECLIN1, PINK1, and PARKIN, alongside decreased expression of P62, TOM20, and COXIV. Moreover, both the PARKIN/COXIV and LC3BII/LC3BI ratios were markedly elevated, indicating that Alisol A and NMN enhance mitophagic activity and facilitate autophagic flux (Figure 7A, *P* < 0.05, *P* < 0.01). Further comparison with the FK866 group revealed that Alisol A and NMN treatment similarly upregulated BECLIN1, PINK1, and PARKIN and downregulated P62, TOM20, and COXIV, while significantly increasing the PARKIN/COXIV and LC3BII/LC3BI ratios, suggesting that NAMPT inhibition impairs autophagic flux (Figure 7A, *P* < 0.05, *P* < 0.01).

The PARKIN/COXIV ratio is used to assess mitophagy activation 111. Recruitment of PARKIN signifies recognition of damaged mitochondria and initiation of their clearance, whereas COXIV-a mitochondrial membrane protein-reflects overall mitochondrial abundance 112, 113. An increased ratio indicates more efficient PARKIN targeting of mitochondria and enhanced mitophagic activity; a decreased ratio suggests impaired targeting or blockade of the autophagic process. Thus, this ratio serves as a dynamic marker for monitoring selective mitophagy. In our *in vitro* experiments, we employed two cellular models to evaluate NAMPT's regulation of autophagy: (1) HT22 cells exposed to Aβ_25-35_ plus cholesterol complex, and (2) primary mouse neurons co-incubated with ox-LDL to mimic lipotoxic injury. Autophagy-related protein levels were then measured by Western blot. Compared with the NAMPT-overexpression group, FK866 treatment alone led to increased P62, TOM20, and COXIV expression, while BECLIN1, PINK1, PARKIN, the PARKIN/COXIV ratio, and the LC3BII/LC3BI ratio all decreased, indicating a blockade of autophagic flux (Figure 7B, *P* < 0.05, *P* < 0.01). In HT22 cells specifically, FK866 treatment-relative to Alisol A-upregulated P62 and COXIV and downregulated BECLIN1, PINK1, and PARKIN, with concomitant reductions in both the PARKIN/COXIV and LC3BII/LC3BI ratios, further supporting NAMPT inhibition-induced impairment of mitophagic flux (Figure 7B, *P* < 0.05, *P* < 0.01). Finally, compared with FK866 alone, combined treatment with Alisol A and FK866 resulted in decreased COXIV expression and an increased PARKIN/COXIV ratio, while P62 expression was elevated; TOM20, PARKIN, and the LC3BII/LC3BI ratio showed no significant change (Figure 7B, *P* < 0.05, *P* < 0.01). In primary neurons, ox-LDL treatment significantly increased P62, TOM20 and COXIV expression while decreasing BECLIN1, PINK1, PARKIN, the PARKIN/COXIV ratio and the LC3BII/LC3BI ratio compared with controls. Relative to the ox-LDL group, NAMPT overexpression markedly lowered P62, TOM20 and COXIV levels and elevated BECLIN1, PINK1, PARKIN, as well as the PARKIN/COXIV and LC3BII/LC3BI ratios, indicating a pivotal role for NAMPT in modulating autophagy and mitochondrial homeostasis (Figure 7B, *P* < 0.05, *P* < 0.01). Conversely, FK866 treatment in ox-LDL exposed neurons further increased P62, TOM20 and COXIV expression and decreased BECLIN1 and the PARKIN/COXIV ratio, whereas PINK1, PARKIN and the LC3BII/LC3BI ratio remained unchanged (Figure 7B, *P* < 0.05, *P* < 0.01). These findings suggest that FK866 inhibits autophagy activation. Despite elevations in certain markers (P62, TOM20, COXIV) that may reflect compensatory autophagic signaling or increased mitochondrial content, the reductions in BECLIN1 and PARKIN/COXIV ratio, together with unchanged PINK1, PARKIN and LC3B processing, indicate suppression of both autophagy initiation and effective mitophagic execution. The decrease in BECLIN1, a key initiator of autophagy, underscores impaired autophagic flux, and the absence of significant change in the LC3BII/LC3BI ratio further corroborates insufficient enhancement of autophagic activity. Mitochondrial membrane potential assays revealed that, compared with FK866 treated cells, those overexpressing NAMPT or treated with Alisol A exhibited strong red and green fluorescence. In contrast, FK866 treated cells showed very faint red and green fluorescence, indicating an overall decline in MMP activity and a reduction in mitochondrial content, likely due to accumulation of damaged mitochondria resulting from impaired mitophagy (Figure 7C, *P* < 0.05, *P* < 0.01).

OPTN, an autophagy receptor, is recruited to the mitochondrial surface where it promotes clearance of damaged mitochondria by binding autophagy proteins such as LC3 114. Immunofluorescence analysis showed that FK866 treatment led to a lower OPTN/TOM20 fluorescence ratio and reduced colocalization of OPTN with the mitochondrial membrane protein TOM20 compared with NAMPT overexpression and Alisol A treatment, indicating impaired receptor recruitment to mitochondria and inhibition of mitophagy (Figure 7D, *P* < 0.01). In contrast, both NAMPT overexpression and Alisol A treatment increased the OPTN/TOM20 ratio and enhanced OPTN-TOM20 colocalization, suggesting activation of the autophagy pathway. OPTN functions as a downstream adaptor of PINK1 and PARKIN 115, 116. Therefore, NAMPT inhibition appears to block the PINK1/PARKIN selective mitophagy pathway: loss of upstream signaling prevents OPTN from localizing to mitochondria and binding LC3, so damaged mitochondria cannot be sequestered and removed. By contrast, NAMPT overexpression activates the PINK1 and PARKIN pathway, enhances OPTN recruitment to mitochondria, restores autophagic flux, clears damaged mitochondria, and interrupts the vicious cycle of mitochondrial dysfunction and oxidative stress.

To further elucidate the molecular mechanism by which NAMPT regulates mitophagy, we investigated whether NAMPT directly interacts with PINK1 to influence PINK1 expression and stability. Knockdown of NAMPT significantly reduced PINK1 levels in HT22 cells (Figure 7E, *P* < 0.01). Co-immunoprecipitation confirmed an interaction between NAMPT and PINK1 (Figure 7F, *P* < 0.01). Further analysis revealed that PINK1 expression was decreased in HT22 cells treated with Aβ_25-35_ plus cholesterol complex and in primary neurons treated with ox-LDL, compared with the non-IgG control, suggesting that these treatments impair the NAMPT-PINK1 interaction. In contrast, Alisol A treatment restored PINK1 expression relative to the Aβ_25-35_/cholesterol and ox-LDL groups, indicating that Alisol A enhances the interaction between NAMPT and PINK1 (Figure 7F, *P* < 0.01).

To clarify NAMPT's role in redox homeostasis in the nervous system and to determine whether Alisol A exerts antioxidant effects via the NAMPT pathway, we evaluated UCP2 expression and ROS levels. Immunofluorescence in the hippocampal CA3 region showed that, compared with *Ldlr^-/-^* mice, both NMN and Alisol A treatment increased UCP2 expression. Relative to FK866 treatment, NMN and Alisol A also elevated UCP2 levels, indicating that enhanced NAMPT activity improves mitochondrial antioxidant capacity and that Alisol A may upregulate UCP2 via NAMPT activation to mitigate oxidative damage (Figure 7G, *P* < 0.01). Compared with Alisol A, FK866 treatment reduced UCP2 expression, demonstrating that NAMPT inhibition weakens the hippocampal antioxidant barrier, exacerbates oxidative stress, and disrupts redox balance. ROS assays revealed that FK866 treatment significantly increased ROS levels compared with NAMPT overexpression. In contrast, both NAMPT overexpression and Alisol A treatment lowered ROS relative to FK866, indicating relief of oxidative stress. Moreover, combined FK866 and Alisol A treatment also reduced ROS compared with FK866 alone, suggesting that Alisol A can partially reverse ROS accumulation (Figure 7H-I, *P* < 0.01). These findings demonstrate that NAMPT is critical for maintaining neural antioxidant defense and that Alisol A confers protective antioxidant effects by upregulating UCP2 and inhibiting ROS accumulation via the NAMPT pathway.

Compared with *Ldlr^-/-^* mice, FK866 treatment reduced Synapsin-1 and PSD95 levels in the CA1 region and the dentate gyrus of the hippocampus, indicating synaptic loss. In contrast, both NMN and Alisol A treatment increased Synapsin-1 and PSD95 expression in these same regions, suggesting that NMN and Alisol A protect synapses (Figure 7J, *P* < 0.01). Golgi staining showed that FK866 diminished dendritic branching and spine density in hippocampal neurons, whereas NMN enhanced both dendritic arborization and spine density in CA1 neurons. Moreover, neurons from the NMN group exhibited a higher proportion of mature mushroom-shaped spines and an overall increase in spine number (Figure 7K, *P* < 0.05, *P* < 0.01). NMDA receptors are critical excitatory glutamate receptors in the central nervous system, and their functional properties depend largely on the types of NR2 subunits they contain 117. Among these, NR2B and NR2A are the most important and best understood; the balance between their expressions is key to synaptic homeostasis and plasticity 118. Immunofluorescence in HT22 cells revealed that FK866 treatment decreased NR2B expression while increasing NR2A levels, indicative of synaptic inhibition. Overexpression of NAMPT reversed these changes, restoring the NR2B/NR2A balance (Figure 7L, *P* < 0.05, *P* < 0.01).

## Discussion

In this study, Alisol A demonstrated potent lipid-modulating and anti-atherosclerotic effects. It lowered serum triglyceride, total cholesterol, and LDL-cholesterol levels, reduced aortic plaque burden, and attenuated lipid deposition in brain tissue. Oil red O and HE staining, together with immunohistochemistry, confirmed that Alisol A markedly decreased lipid content within atherosclerotic lesions, improved plaque architecture, and suppressed α-smooth muscle actin and CD68 expression, indicating stabilization of plaques, anti-inflammatory activity, and inhibition of lesion progression. Behavioral assays including the Morris water maze, Y-maze, and novel object recognition tests revealed that Alisol A significantly enhanced learning, spatial memory, and recognition abilities in *Ldlr^-/-^* mice, indicating restoration and protection of cognitive function. At the synaptic level, Alisol A not only improved hippocampal synaptic ultrastructure evidenced by increased synaptic vesicle density, longer active zone length, narrower synaptic cleft, and thicker postsynaptic density, but also upregulated key synaptic markers Synapsin-1 and PSD95, suggesting promotion of synaptic remodeling and functional coupling. Golgi staining combined with Sholl analysis showed that Alisol A significantly increased dendritic branching complexity, spine density, and the number of mature mushroom-type spines in CA1 neurons. The selective enhancement of mushroom spines, whose enlarged head morphology expands synapse contact area implies that Alisol A restores cognitive function by remodeling synaptic efficacy rather than merely increasing synapse number.

Disruption of cholesterol metabolism and oxidative stress are key pathological mechanisms in the development of AS related VCI. Alterations in brain cholesterol in humans have been implicated in various neurological and neurodegenerative diseases 12, 13. Brain lipids, such as cholesterol, are critical for maintaining neuronal membrane homeostasis and synaptic functions 22, 23. Direct suppression of cholesterol synthesis impairs neurotransmitter release 24. We selected *Ldlr^-/-^* mice as the model for our research experiment on *Ldlr^-/-^*, as they are widely recognized as one of the most commonly used models for studying atherosclerosis. Cholesterol is synthesized in situ within the brain, where its concentration surpasses that of any other organ. Cholesterol uptake, synthesis, and metabolism are predominantly regulated by the SREBP2/HMGCR pathway of sterol-responsive genes 12, 119. LXRs serve as potent regulators of reverse cholesterol transport, thereby impeding the progression of atherosclerosis in murine models 120, 121. We found that Alisol A significantly modulates the expression of several key metabolic regulators. Specifically, it upregulates CYP46A1, LXR-α, and ABCA1 to promote cholesterol efflux and metabolism, while downregulating SREBP2 and HMGCR to inhibit cholesterol synthesis and uptake. These changes collectively contribute to restoring dynamic cholesterol balance within neurons. Alisol A markedly alleviates oxidative-reductive imbalance in the hippocampus of *Ldlr^-/-^* mice. It upregulates the antioxidant enzymes GSH and SOD and the mitochondrial protein UCP2, while reducing levels of the lipid peroxidation product MDA and ROS. *In vitro*, Alisol A pretreatment significantly suppresses ROS accumulation in HT22 cells. Since UCP2 is a key regulator of ROS, its increased expression lowers mitochondrial membrane potential (ΔΨm), creating a superoxide-mediated negative feedback loop that inhibits complex I activity and reduces ROS production. This mechanism was confirmed in HT22 cells co-treated with Aβ_25-35_ and cholesterol complex, where Alisol A pretreatment restored ROS fluorescence intensity to baseline. Furthermore, Alisol A downregulates GFAP in astrocytes and IBA1 in microglia. Sholl analysis shows reduced glial branching complexity and activation, indicating an anti-inflammatory effect. Structurally, Alisol A improves neuronal morphology in the CA1 and dentate gyrus regions of *Ldlr^-/-^* mouse hippocampi: it decreases the number of degenerating neurons and increases Nissl body density, confirming its robust neuroprotective effects at both the structural and functional levels. In summary, Alisol A regulates cholesterol metabolism pathways, upregulates UCP2 expression to alleviate oxidative stress, and inhibits glial cell activation, thereby preserving neuronal structure and function.

Mendelian randomization analysis confirmed that atherosclerosis is a significant risk factor for vascular dementia. Combined lipid metabolomics revealed that differential metabolites were significantly enriched in the AMPK signaling pathway and autophagy pathways. Analysis of GEO datasets showed that NAMPT expression was most markedly altered in atherosclerosis patient samples (*P* < 0.01). To validate the mechanism, we performed molecular docking, molecular dynamics simulation, and surface plasmon resonance assays, demonstrating that Alisol A binds stably to NAMPT by forming hydrogen bonds and hydrophobic contacts at Gln 305 and Tyr 188, with a binding affinity of KD = 65.1 μM. *In vivo*, Alisol A treatment significantly upregulated p-AMPKα, NAMPT, SIRT1, and PGC-1α expression, increased NAD⁺ levels, reduced acetyl-lysine modifications, and activated the AMPK/NAMPT/SIRT1 axis. In HT22 cells exposed to Aβ_25-35_ plus cholesterol complex and in primary neurons treated with ox-LDL, Alisol A reversed suppression of the AMPK/NAMPT/SIRT1 axis and NAD⁺ depletion, confirming its positive regulation of cellular energy homeostasis and antioxidant defense. NAMPT plays a critical role in cellular energy homeostasis and mitophagy by regulating NAD⁺ biosynthesis and SIRT1 activity 122-124. AMPK serves as an energy sensor that initiates metabolic responses, while NAMPT functions as the rate-limiting enzyme in NAD⁺ regeneration, providing essential metabolic support for AMPK-dependent activation of SIRT1 125-127. Together, these findings indicate that Alisol A activates AMPK/NAMPT/SIRT1 signaling and elevates NAD⁺ levels, providing a potential target and molecular basis for intervening in AS related VCI.

Lysine acetylation represents a reversible post-translational modification capable of modifying protein conformation, consequently influencing its stability, DNA binding affinity, and enzymatic activity. This modification is intricately linked to the cellular metabolic state, as acetyl CoA, the primary substrate for energy production, serves as the exclusive donor of acetyl groups for acetylation processes. Additionally, NAD^+^ functions as a pivotal metabolite for energy and redox homeostasis and acts as a cofactor for sirtuin (SIRT) deacetylases. Protein acetylation has emerged as a pivotal regulatory mechanism for autophagy 128. Many proteins functioning at different stages of the autophagy pathway have been identified as targets of acetylation. The initiation of autophagy is intricately regulated by acetylation processes involving key molecular components. The mechanistic target of rapamycin kinase complex 1 (MTORC1) and AMPK are pivotal in modulating autophagy initiation. SIRT1, a deacetylase, plays a crucial role by inhibiting acetylation, thereby facilitating the control of autophagy initiation. As the major deacetylase functioning in autophagy, SIRT1 can be activated through an AMPK dependent manner upon glucose deprivation. Activated SIRT1 then deacetylates nuclear LC3 to facilitate autophagy initiation. These findings highlight the crucial role of *sirt1*- mediated *nampt* deacetylation on *Ldlr^-/-^*, suggesting targeting *nampt* has the potential to reverse AS-induced autophagy deficiency and provide effective treatment options for *Ldlr^-/-^*. *Sirt1*, a NAD⁺-dependent deacetylase, promotes autophagy by deacetylating key autophagy-related proteins such as Atg5, Atg7, and Atg8 102, 103. These deacetylation events are essential for the initiation and maintenance of autophagic activity. *Nampt* regulates intracellular NAD⁺ levels and thereby indirectly activates *sirt1*, further influencing autophagy processes 129, 130. The activation of autophagy relies on deacetylation of multiple critical autophagy regulators. *Sirt1* enhances autophagy initiation by targeting lysine residues on autophagy-related proteins for deacetylation 88, 131.

In this study, *in vivo* experiments showed that administration of the *nampt*-specific inhibitor FK866 significantly exacerbated cognitive impairment in *Ldlr^-/-^* mice. Behavioral tests revealed prolonged escape latency in the Morris water maze, reduced spontaneous alternation in the Y-maze, and impaired novel object recognition. Concurrently, FK866 markedly suppressed the expression of NAMPT and SIRT1 in the hippocampus and reduced NAD⁺ levels. *In vitro*, FK866 upregulated the expression of cholesterol synthesis-related factors SREBP2 and HMGCR, while downregulating LXR-α and inducing a reduction in hippocampal CYP46A1 expression. These changes led to cholesterol accumulation and metabolic imbalance. Similarly, NAMPT knockdown or FK866 treatment increased the expression of SREBP2 and HMGCR, while suppressing LXR-α, ABCG5, ABCG8, and SIRT1 expression, accompanied by elevated levels of lysine acetylation-further confirming NAMPT's regulatory role in cholesterol metabolism. Immunohistochemical staining of hippocampal tissue corroborated these findings, showing that CYP46A1 expression was significantly downregulated following FK866 treatment, whereas both Alisol A and NMN reversed this reduction. COIP assays demonstrated a direct or indirect protein-protein interaction between NAMPT and CYP46A1. NAMPT overexpression enhanced this interaction, while it was notably weakened in HT22 cells treated with Aβ_25-35_ and cholesterol complexes, as well as in ox-LDL exposed primary neurons. These results suggest that high-cholesterol and stress conditions disrupt NAMPT-CYP46A1 coupling. Notably, Alisol A treatment effectively restored the NAMPT-CYP46A1 interaction, indicating that Alisol A may exert lipid-regulatory and neuroprotective effects by enhancing the functional coupling between NAMPT and CYP46A1, thereby co-regulating cholesterol uptake and efflux pathways. In the brain, cholesterol is a critical component of neuronal function 25, 26, and its dysregulation is closely associated with various neurodegenerative diseases 23, 27, 132, 133. *Cyp46a1* is a brain-specific cholesterol-metabolizing enzyme 19, 28, 29. The suppression of CYP46A1 expression by FK866 indicates that NAMPT plays a vital role in maintaining cerebral cholesterol homeostasis. Disruption of this pathway may lead to cholesterol accumulation in the brain, further contributing to cognitive decline. Altogether, these findings underscore the crucial role of NAMPT in regulating cholesterol metabolism and cognitive function. Modulating NAMPT activity may offer a novel therapeutic strategy for treating neurodegenerative diseases associated with cholesterol dysregulation.

Mitophagy is essential for maintaining mitochondrial homeostasis by selectively degrading damaged mitochondria, thus adjusting mitochondrial quantity in response to metabolic demands 134, 135. Notably, mitophagy preserves a healthy mitochondrial pool and prevents neuronal death, making it critical for neuronal function and survival 111. Enhancing autophagy has been shown to mitigate neurological dysfunction 111, 136, and autophagy inducers have demonstrated neuroprotective effects in mouse models 137-139. Regulating the PINK1-mediated mitophagy pathway can improve mitochondrial dysfunction and ameliorate cognitive deficits 111, 140. Previous studies have reported that Alisol A can induce autophagy in a dose-dependent manner 49, 50. Consistent with these findings, our results show that Alisol A treatment significantly increased LC3BII/LC3BI and BECLIN1 levels, while reducing P62 and TOM20 expression, indicating enhanced autophagic flux. In addition, elevated levels of PINK1 and the PARKIN/COXIV ratio were observed, which may reflect Alisol A induced recruitment of PARKIN to damaged mitochondria via PINK1 activation. This recruitment promotes LC3B engagement and triggers PINK1/PARKIN dependent mitophagy, leading to TOM20 degradation and a reduction in mitochondrial COXIV protein levels. Taken together, these findings demonstrate that Alisol A markedly improves mitophagy function by activating the AMPK/NAMPT/SIRT1 signaling pathway in hippocampal tissue and neuronal cells. In Aβ_25-35_ plus cholesterol complex treated cellular models and in ox-LDL exposed primary neurons, Alisol A was found to activate the PINK1/PARKIN mediated mitophagy pathway, facilitating the recognition and clearance of damaged mitochondria and thereby restoring mitochondrial function. Furthermore, Alisol A treatment improved mitochondrial membrane potential and enhanced autophagic flux, supporting its role in restoring mitochondrial homeostasis and improving cognitive function. Based on these findings, this study suggests that Alisol A may improve cognitive impairment in *Ldlr^-/-^* mice by maintaining mitophagy homeostasis. Notably, although NAMPT was identified as a key regulator of mitophagy, we further observed that Alisol A was still able to partially restore the PINK1/PARKIN signaling pathway, enhance LC3B conversion, and reduce P62 expression under conditions of NAMPT inhibition. This indicates that Alisol A possesses a degree of NAMPT-independent regulatory activity on autophagy. Importantly, this observation does not diminish the role of NAMPT in mitophagy regulation. Instead, it highlights the multi-target and multi-mechanism pharmacological nature of Alisol A, revealing its potential to preserve mitochondrial integrity even under complex pathological conditions such as NAMPT suppression or NAD⁺ depletion. This study not only deepens our understanding of NAMPT's role in neuronal homeostasis but also provides important theoretical support for the therapeutic application of Alisol A in AS related VCI.

In this study, it was noted that an elevation in the autophagy substrate protein P62, in conjunction with PINK1/PARKIN-dependent compromised mitophagy, was apparent in *Ldlr^-/-^*, resulting in neuronal degeneration in the hippocampus. This study's findings indicate that disruption of mitophagy and mitochondrial dynamics, resulting from mitochondrial dysfunction, are critical mechanisms underlying cholesterol-induced synaptic impairment and cognitive decline in the hippocampus. Treatment with Alisol A significantly elevates LC3BII/LC3BI and Beclin1 levels, while decreasing P62 and TOM20 levels, indicating that Alisol A enhances autophagic flux. Additionally, the observed increases in PINK1 and PARKIN/COXIV levels. This might be interpreted as treatment with Alisol A stimulates PINK1-induced translocation of Parkin to damaged mitochondria the latter links LC3B to initiate mitophagy, namely, the PINK1/PARKIN-dependent mitophagy pathway to degrade TOM20 and induces a decrease in mitochondrial COXIV protein level. Our results showed that Alisol A treatment can more effectively prevent mitochondrial damage and autophagy inhibition in HT22 exposed to Aβ_25-35_ and cholesterol complexes. NAMPT knockout reduced PINK1 expression in HT22 cells. Our COIP assay shows that NAMPT interacts with PINK1, and Alisol A -NAMPT binding boosts PINK1 activity, enhancing autophagy by increasing PARKIN recruitment. Based on these results, we believe that Alisol A may ameliorate *Ldlr^-/-^* by maintaining the homeostasis of mitophagy.

Immunofluorescence analysis showed that FK866 significantly reduced UCP2 expression, while NMN and Alisol A treatment restored UCP2 levels. ROS assays revealed that FK866 increased ROS accumulation, whereas NAMPT overexpression and Alisol A treatment effectively reduced ROS levels. At the synaptic level, FK866 decreased Synapsin-1 and PSD95 expression in the hippocampus, as shown by immunofluorescence, while both NMN and Alisol A upregulated these synaptic markers. Golgi staining combined with Sholl analysis confirmed that Alisol A increased dendritic spine density and the proportion of mature mushroom-shaped spines, ameliorating FK866-induced dendritic damage. Analysis of NMDA receptor subunits revealed that FK866 suppressed NMDA 2B and increased NMDA 2A expression, indicating a shift toward synaptic inhibition. Alisol A restored the NMDA 2B/2A expression ratio, suggesting enhanced synaptic signaling. Collectively, these findings demonstrate that NAMPT plays a key role in regulating neuronal oxidative stress and synaptic plasticity. Upregulation of NAMPT helps maintain synaptic structure and functional stability. By activating the NAMPT pathway, Alisol A alleviates oxidative damage and promotes synaptic remodeling, thereby effectively improving VCI-related neurological dysfunction. In conclusion, NAMPT is a critical therapeutic target of Alisol A in the treatment of atherosclerosis-related vascular cognitive impairment. Through NAMPT regulation, Alisol A improves cholesterol metabolism, restores mitophagic activity, facilitates the clearance of damaged mitochondria, reduces oxidative stress, and enhances synaptic plasticity, ultimately exerting neuroprotective effects.

However, this study has certain limitations. Although FK866 induces MMP collapse and activates apoptosis by inhibiting NAMPT, it lacks single-cell level tracking of the spatiotemporal dynamics underlying the crosstalk between autophagy and apoptosis. It is important to note that autophagy plays a dual role in neurodegenerative conditions: moderate autophagy can remove misfolded proteins and support neuronal survival, whereas excessive autophagy may lead to lysosomal membrane permeabilization and trigger apoptosis 141. Future studies should employ CRISPR interference (CRISPRi) systems to achieve temporally controlled regulation of autophagic flux in specific brain regions 142-144, and integrate two-photon imaging to explore the dynamic interplay between dendritic spine remodeling and apoptotic signaling 145.

## Conclusions

In conclusion, this study demonstrates two key findings: First, Alisol A exerts neuroprotective effects by activating the AMPK/NAMPT/SIRT1 signaling pathway in hippocampal tissue and neurons, thereby promoting PINK1/PARKIN-mediated mitophagy and restoring the function of damaged mitochondria. Second, NAMPT is identified as a critical therapeutic target of Alisol A in the treatment of AS related VCI. Through regulation of NAMPT, Alisol A improves cholesterol metabolism, restores mitophagic activity, facilitates the clearance of damaged mitochondria, alleviates oxidative stress, and promotes the reconstruction of synaptic plasticity, ultimately contributing to neuroprotection. Our research results offer valuable insights for the continued advancement of Alisol A as a promising lead compound in research as potential anti-AS related VCI candidates.

## Supplementary Material

Supplementary tables.

## Abbreviations

AS: Atherosclerosis
VCI: Vascular cognitive impairment
ZXYF: ZeXieYin Formula
VaD: Vascular dementia
LDL: Low-density lipoprotein
LDL-c: Low-density lipoprotein cholesterol
ox-LDL: Oxidized low-density lipoprotein
CSVD: Cerebral small vessel disease
BBB: Blood brain barrier
Aβ: Amyloid-β
LXR: Liver X receptor
NLRP3: NOD-like receptor family pyrin domain containing 3
TCM: Traditional Chinese medicine
AMPK: AMP-activated protein kinase
NAMPT: Nicotinamide phosphoribosyltransferase
SIRT1: Sirtuin 1
NMN: Nicotinamide mononucleotide
MWM: Morris water maze
NOR: Novel object recognition
ROS: Reactive oxygen species
SOD: Superoxide dismutase
GSH: Glutathione
MDA: Malondialdehyde
UCP2: Uncoupling protein 2
CYP46A1: Cholesterol 24-hydroxylase
HMGCR: 3-hydroxy-3-methylglutaryl-coenzyme A reductase
ABCA1: ATP-binding cassette transporter A1
ABCG5/8: ATP-binding cassette subfamily G members 5/8
SREBP2: Sterol regulatory element-binding protein 2
PGC1-α: Peroxisome proliferator-activated receptor gamma coactivator 1-alpha
LC3B: Microtubule-associated protein 1A/1B-light chain 3B
PINK1: PTEN-induced kinase 1
PARKIN: E3 ubiquitin-protein ligase PARK2
TOM20: Translocase of the outer membrane 20
COXIV: Cytochrome c oxidase subunit IV
BECLIN1: Autophagy protein Beclin-1
P62/SQSTM1: Sequestosome 1
OPTN: Optineurin
NR2A/NR2B: NMDA receptor subunits 2A and 2B
PSD95: Postsynaptic density protein 95
SPR: Surface plasmon resonance
GEO: Gene Expression Omnibus
GWAS: Genome-wide association study
IVW: Inverse variance weighted
TEM: Transmission electron microscopy
IHC: Immunohistochemistry
IF: Immunofluorescence
COIP: Co-immunoprecipitation
MMP: Mitochondrial membrane potential
TSA: Trichostatin A
NAM: Nicotinamide
NAD⁺: Nicotinamide adenine dinucleotide
