# Supplementary material for: Alisol A ameliorates vascular cognitive impairment via AMPK/NAMPT/SIRT1-mediated regulation of cholesterol and autophagy
Source: Theranostics. 2025 Aug 22;15(18):9415–46. doi: 10.7150/thno.112661 (PMC12486161; doi:10.7150/thno.112661)
Supplement: Supplementary file 1 — Supplementary tables. [file thnov15p9415s1.pdf]

Table S1 Statistics of testing for heterogeneity in Mendelian randomization analysis

exposure	outcome	method	Q	Q_df	Q_pval
finn-b-I9_CORATHER	finn-b-F5_VASCDEM	Inverse variance weighted	30.833	29	3.73e-1
finn-b-I9_CORATHER	finn-b-F5_VASCDEM	MR Egger	30.533	28	3.38e-1

Table S2 Statistics of testing for pleiotropy in Mendelian randomization analysis

In this study, the term "Exposure" refers to the numerical data associated with the exposure variable, while "Outcome" denotes the numerical data pertaining to the outcome variable. The "Egger_intercept" represents the intercept obtained from the Egger regression analysis, and "SE" indicates the standard error associated with this intercept. The "PAL" refers to the p-value of the Egger regression intercept. A p-value less than the predetermined significance level (commonly set at 0.05) suggests the presence of pleiotropy within the dataset.

exposure	outcome	Egger_intercept	se	pval
finn-b-I9_CORATHER	finn-b-F5_VASCDEM	0.020	0.038	6.04e-01

Table S3 Mendelian randomization analysis

This table displays the Mendelian randomization estimates for the causal effects of exposure on outcomes, categorized by each methodological approach.

In this study, "Exposure" and "Outcome" refer to the numerical data for their respective variables. "Method" describes the model used for Mendelian randomization analysis, and "Nsnp" is the count of single nucleotide polymorphisms (SNPs) used. The symbol "b" denotes the regression coefficient beta, indicating the strength and direction of the relationship between exposure and outcome variables. A beta greater than zero suggests a consistent direction, while a beta less than zero indicates opposite directions. The Standard Error (SE) measures the variability of the regression coefficients and helps evaluate the statistical significance of the relationship. If p<0.05, a causal relationship is suggested; OR: Odds ratio or dominance ratio. An OR value of 1 means the factor doesn't affect disease risk. An OR greater than 1 suggests increased risk, while an OR less than 1 indicates reduced risk. Or_1ci95 and Or_uci95 represent the lower and upper limits of the 95% confidence interval for OR values, respectively.

**exposure**	**outcome**	**method**	**nsnp**	**b**	**se**	**pval**	**or**	**or_lci95**	**or_uci95**
finn-b-I9_CORATHER	finn-b-F5_VASCDEM	MR Egger	30	0.057	0.317	8.57e-01	1.059	0.569	1.971
finn-b-I9_CORATHER	finn-b-F5_VASCDEM	Weighted median	30	0.175	0.148	2.37e-01	1.191	0.891	1.592
finn-b-I9_CORATHER	finn-b-F5_VASCDEM	Inverse variance weighted	30	0.214	0.107	4.48e-02	1.238	1.005	1.526
